# Impact of stress on cardiac phenotypes in mice harboring an ankyrin-B disease variant

**DOI:** 10.1016/j.jbc.2023.104818

**Published:** 2023-05-12

**Authors:** Michael J. Wallace, Nipun Malhotra, Juan Ignacio Elio Mariángelo, Tyler L. Stevens, Lindsay J. Young, Steve Antwi-Boasiako, Danielle Abdallah, Sarah Sumie Takenaka, Omer Cavus, Nathaniel P. Murphy, Mei Han, Xianyao Xu, Matteo E. Mangoni, Thomas J. Hund, Jason D. Roberts, Sandor Györke, Peter J. Mohler, Mona El Refaey

**Affiliations:** 1 The Frick Center for Heart Failure and Arrhythmia, Dorothy M. Davis Heart and Lung Research Institute, The Ohio State University, Columbus, Ohio, USA; 2 Department of Physiology and Cell Biology, The Ohio State University, Columbus, Ohio, USA; 3 Department of Surgery/Division of Cardiac Surgery, The Ohio State University, Columbus, Ohio, USA; 4 Institut de Génomique Fonctionnelle, Université de Montpellier, CNRS, INSERM, Montpellier, France; 5 Department of Biomedical Engineering, College of Engineering, The Ohio State University, Columbus, Ohio, USA; 6 Department of Internal Medicine/Division of Cardiovascular Medicine, The Ohio State University, Columbus, Ohio, USA; 7 Population Health Research Institute, McMaster University, and Hamilton Health Sciences, Hamilton, Ontario, Canada

**Keywords:** arrhythmia, ankyrin-b, bradycardia, cardiovascular disease, fibrosis, heart rate variability, heart failure, incomplete penetrance, stress, variant

## Abstract

Encoded by *ANK2*, ankyrin-B (AnkB) is a multifunctional adapter protein critical for the expression and targeting of key cardiac ion channels, transporters, cytoskeletal-associated proteins, and signaling molecules. Mice deficient for AnkB expression are neonatal lethal, and mice heterozygous for AnkB expression display cardiac structural and electrical phenotypes. Human *ANK2* loss-of-function variants are associated with diverse cardiac manifestations; however, human clinical ‘AnkB syndrome’ displays incomplete penetrance. To date, animal models for human arrhythmias have generally been knock-out or transgenic overexpression models and thus the direct impact of *ANK2* variants on cardiac structure and function *in vivo* is not clearly defined. Here, we directly tested the relationship of a single human *ANK2* disease-associated variant with cardiac phenotypes utilizing a novel *in vivo* animal model. At baseline, young AnkBp.E1458G^+/+^ mice lacked significant structural or electrical abnormalities. However, aged AnkBp.E1458G^+/+^ mice displayed both electrical and structural phenotypes at baseline including bradycardia and aberrant heart rate variability, structural remodeling, and fibrosis. Young and old AnkBp.E1458G^+/+^ mice displayed ventricular arrhythmias following acute (adrenergic) stress. In addition, young AnkBp.E1458G^+/+^ mice displayed structural remodeling following chronic (transverse aortic constriction) stress. Finally, AnkBp.E1458G^+/+^ myocytes harbored alterations in expression and/or localization of key AnkB-associated partners, consistent with the underlying disease mechanism. In summary, our findings illustrate the critical role of AnkB in *in vivo* cardiac function as well as the impact of single AnkB loss-of-function variants *in vivo.* However, our findings illustrate the contribution and in fact necessity of secondary factors (aging, adrenergic challenge, pressure-overload) to phenotype penetrance and severity.

Ankyrin-B (AnkB), encoded by *ANK2*, was first identified to play a role in the nervous system *via* maintenance of premyelinated axons (1) and subsequently determined to impact cardiac function. AnkB regulates the expression and targeting of key membrane, cytoskeletal, and regulatory proteins, including the Na^+^/Ca^2+^ exchanger (NCX), Na^+^/K^+^-ATPase (NKA), voltage-gated calcium channel (Ca_v_1.3), protein phosphatase 2 A, and β-spectrin (2, 3). *ANK2* encodes multiple isoforms and is subject to transcriptional regulation (4).

AnkB dysfunction is associated with diverse human cardiac phenotypes, including QT prolongation, arrhythmogenic cardiomyopathy (ACM), sinus node dysfunction, and atrial fibrillation. Rare *ANK2* variants may also function as modifiers of wall thickness in hypertrophic cardiomyopathy (5). The broad spectrum of AnkB molecular partners in the heart likely accounts for the corresponding broad range of cardiac phenotypes associated with *ANK2* dysfunction. The constellation of clinical features observed with *ANK2* dysfunction has been termed ‘AnkB syndrome’ (6, 7, 8, 9, 10).

The human AnkBp.Glu1458Gly variant is associated with arrhythmias and structural remodeling (7). However, AnkB syndrome displays incomplete penetrance, suggesting that AnkB variants alone may not be sufficient to directly cause arrhythmia and/or structural phenotypes. Mouse gene KO models may not replicate human molecular phenotypes (*e.g.* in case of dominant-negative variants). Therefore, we engineered a new animal model harboring the AnkB variant p.E1458G. Here, we illustrate two points: 1, single AnkB variants are sufficient to cause cardiac phenotypes in 6-month-old AnkBp.E1458G^+/+^ mice and 2, acute and/or chronic stress augments the genesis of cardiac disease phenotypes in both young and old mice carrying the human variant. Our findings support a role of single *ANK2* variants in promoting mild cardiac phenotypes *in vivo*. However, severe *in vivo* phenotypes require secondary stressors including aging and adrenergic stress.

## Results

### Generation of a novel humanized knock-in mouse model

**Figure 1:**
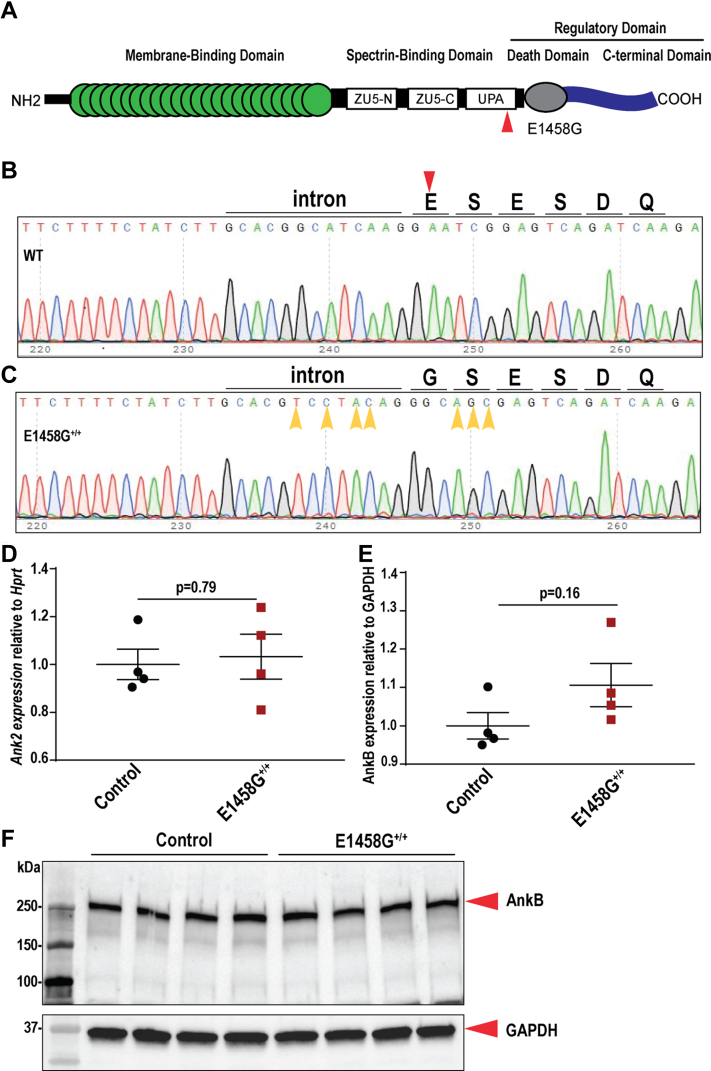
**Creation of a novel AnkBp.E1458G knock-in mouse model.***A*, diagrammatic illustration denoting the location of the variant in the spectrin-binding domain of canonical ankyrin-B. *B* and *C*, representative Sanger sequencing chromatogram denoting the amino acid sequence in the control and the AnkBp.E1458G^+/+^ mice and confirming the substitution of the glutamic acid (E) by glycine (G). *D*, *Ank2* relative expression in control and AnkBp.E1458G^+/+^ mice at 7 weeks of age (N = 4 mice/genotype); data passed Shapiro–Wilk normality test, and unpaired *t* test was performed. *E* and *F*, immunoblotting and quantitative analysis of normalized AnkB expression in the control and the AnkBp.E1458G^+/+^ mice (N = 4 mice/genotype); data passed Shapiro–Wilk normality test, and unpaired *t* test was performed. *Red arrow* denotes the change in the amino acid. *Yellow arrows* denote the multiple silent changes to block the Cas9 targets. AnkB, ankyrin-B.

The AnkBp.E1458G variant (Fig. 1*A*) is associated with AnkB syndrome (3) and ACM (7). To test if this variant is sufficient to induce cardiac phenotypes *in vivo*, we generated a homozygous knock-in (KI) model (Fig. 1, *B* and *C*) harboring this single amino acid substitution. Cas9 was used to introduce a double strand chromosome break near codon 1371 in mouse (corresponds to 1458 in human sequence, ENSMUST00000182078.9). A single stranded oligonucleotide donor was used to change E1371 (E1458) to G1371 (G1458), and multiple silent coding changes were also introduced to block the Cas9 targets. Sanger sequencing chromatograms were used to confirm the substitution of glutamic acid (E) by glycine (G) in the KI mouse (Fig. 1, *B* and *C*). AnkBp.E1458G^+/+^ mice were born at normal Mendelian ratios, 29% control mice (40/137), 46% heterozygous mice (63/137), and 25% homozygous mice (34/137). *Ank2* mRNA expression levels were not changed in hearts of AnkBp.E1458G^+/+^ mice compared to control littermates (Fig. 1*D*). Canonical AnkB protein levels were not significantly different between AnkBp.E1458G^+/+^ and control littermate hearts (Fig. 1, *E* and *F*).

### Young AnkBp.E1458G^+/+^ mice do not display cardiac phenotypes

**Figure 2:**
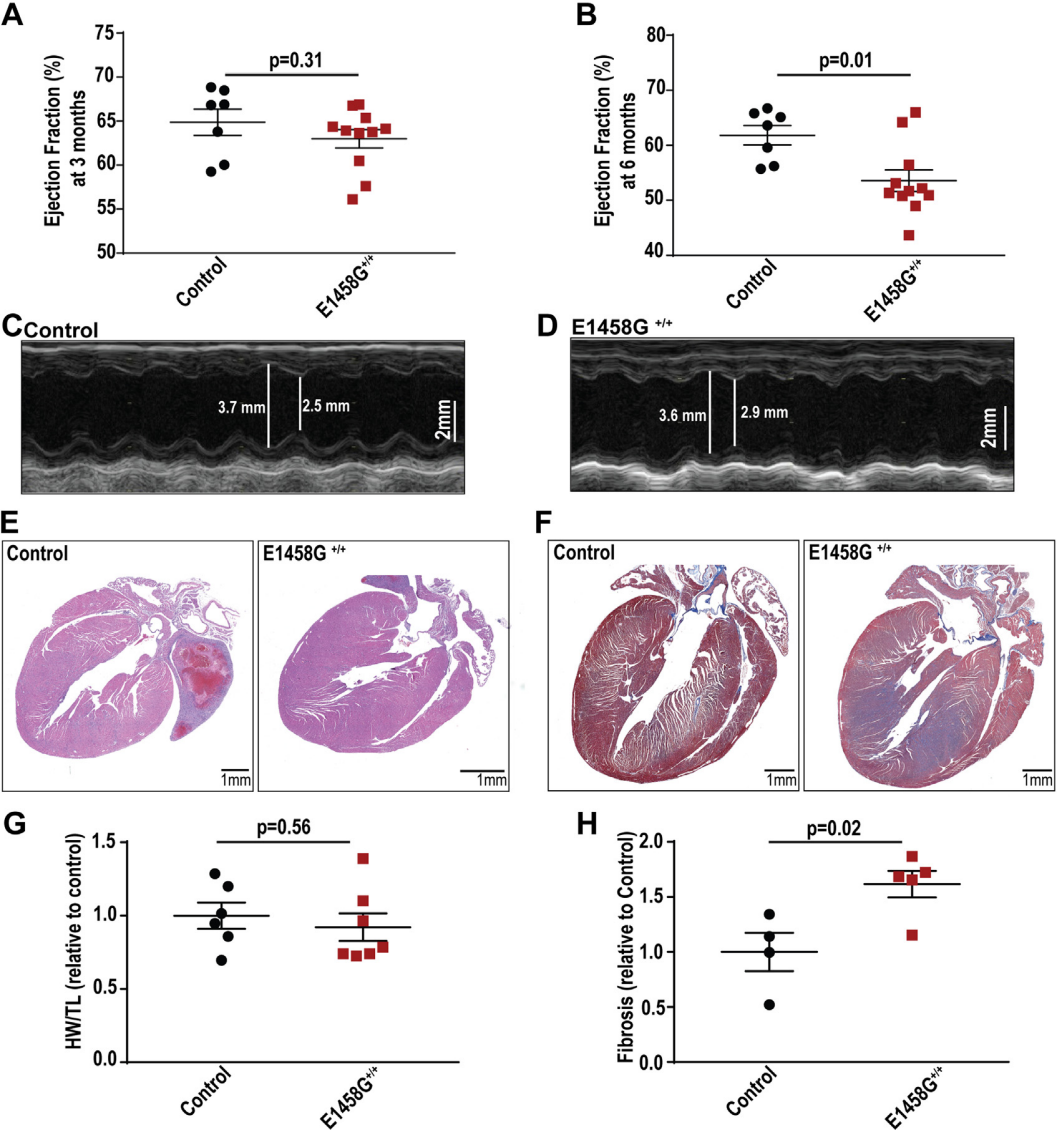
**AnkBp.E1458G**^**+/+**^**mice display a cardiac phenotype at ∼6 months of age.***A*, control and AnkBp.E1458G^+/+^ mice do not display changes in the ejection fraction around 3 months of age (young mice); data passed Shapiro–Wilk normality test, and unpaired *t* test was performed. *B*–*D*, quantitative analysis and representative echocardiographs denoting a reduction in ejection fraction in the AnkBp.E1458G^+/+^*versus* the control littermates at ∼6 months of age (con, N = 7 and AnkBp.E1458G^+/+^, N = 11). Scale bars represent 2 mm. Data passed Shapiro–Wilk normality test, and unpaired *t* test was performed. *E*, H&E staining and (*F*) Masson’s trichrome staining using control and AnkBp.E1458G^+/+^ heart sections in aged mice con, N = 4 and AnkBp.E1458G^+/+^, N = 5). *G*, heart weight relative to tibial length in the two groups of mice (con, N = 6 and AnkBp.E1458G^+/+^, N = 7, around 5 months of age). Data passed Shapiro–Wilk normality test, and unpaired *t* test was performed. *H*, quantitative analysis of fibrosis (con, N = 4 and AnkBp.E1458G^+/+^, N = 5, around 5 months of age). Scale bars represent 1 mm. Data passed Shapiro–Wilk normality test, and unpaired *t* test was performed. AnkB, ankyrin-B.

Three-month-old AnkBp.E1458G^+/+^ homozygous mice did not display structural phenotypes. Specifically, 3-month-old AnkBp.E1458G^+/+^ mice did not display changes in ejection fraction or fractional shortening (Figs. 2*A* and S1, *A*, *G* and *H*). Moreover, AnkBp.E1458G^+/+^ mice did not exhibit electrocardiogram alterations or arrhythmia phenotypes at 3 months of age (Fig. S2).

### Aged AnkBp.E1458G^+/+^ mice display structural remodeling and reduced cardiac function

AnkBp.E1458G^+/+^ mice showed a significant reduction in ejection fraction and fractional shortening at ∼6 months of age (Figs. 2, *B*–*D*, S1*B* and Table S1). Six-month-old AnkBp.E1458G^+/+^ mice also exhibited a trend towards an increased left ventricular internal diameter (LVID, *p* = 0.0694, Fig. S1*C*) and decreased left ventricular posterior wall (LVPW) during systole (*p* = 0.0971, Fig. S1*D*) that did not reach statistical significance. No significant changes were noted in LVID or LVPW during diastole (Fig. S1, *E* and *F*). While *Ank2*-cKO mice showed left ventricular dilation and increased body weight relative to tibial length (7), these findings were not observed in the AnkBp.E1458G^+/+^ mice in this study (Fig. 2, *E*–*G*). Notably, 6-month-old AnkBp.E1458G^+/+^ mice showed widespread cardiac fibrosis at baseline compared with control mice (Fig. 2, *F* and *H*). In summary, aged AnkBp.E1458G^+/+^ mice developed structural remodeling characterized by cardiac fibrosis and reduced cardiac function in the absence of stress. No significant changes were noted in the cardiac remodeling markers including β-myosin heavy chain (*Myh7*), atrial natriuretic peptide (*Nppa*), brain natriuretic peptide (*Nppb*), collagen type I alpha 1 chain (*Col1a1*), and tissue inhibitor matrix metalloproteinase 1 (*Timp1*) in the AnkBp.E1458G^+/+^ mice at ∼ 6 months of age (Fig. S3).

### Aged AnkBp.E1458G^+/+^ mice exhibit electrical phenotypes

**Figure 3:**
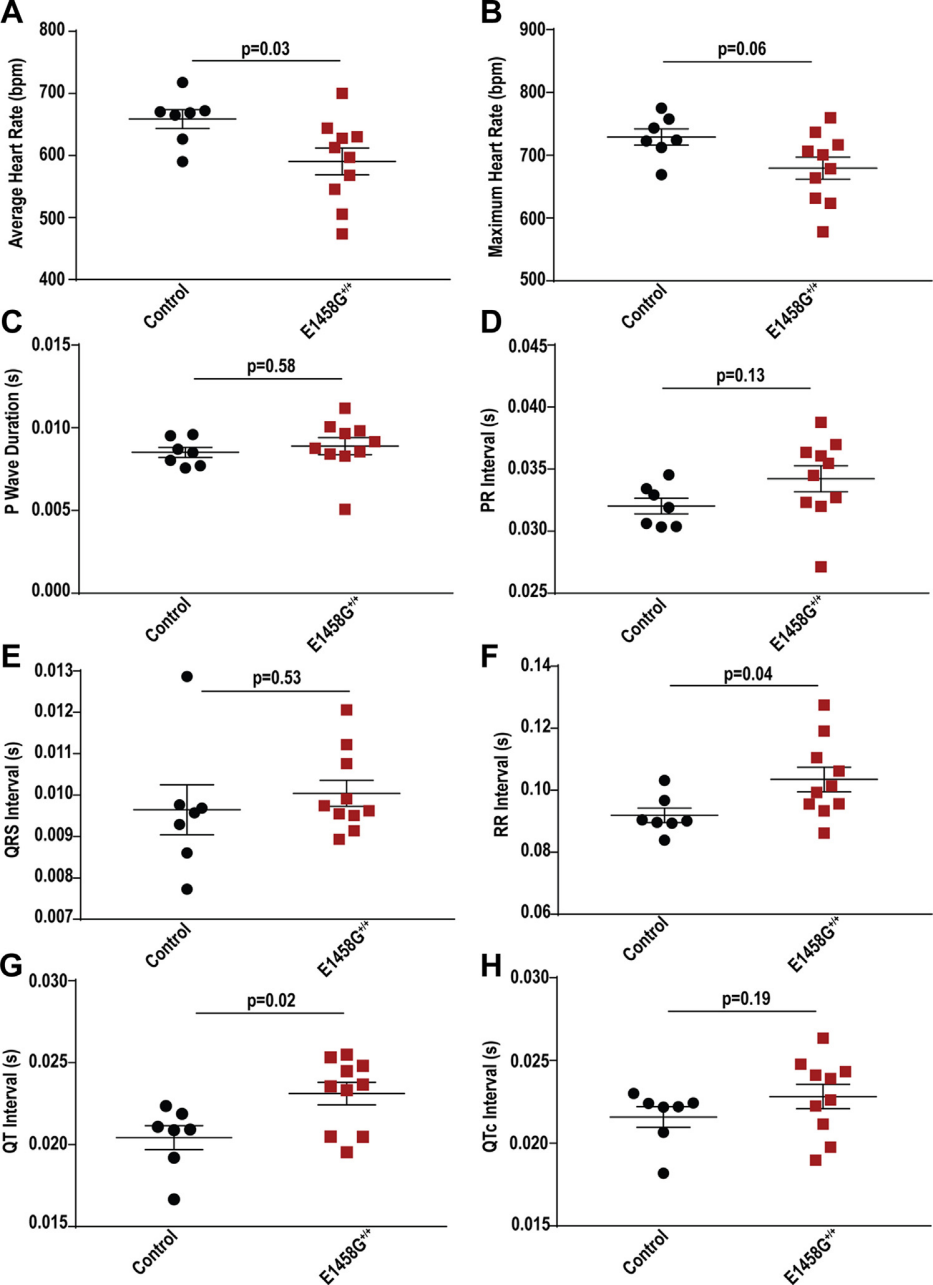
**Aged AnkBp.E1458G**^**+/+**^**mice display electrical dysfunction at rest.***A*, average heart rate, (*B*) maximal heart rate, (*C*) P wave duration, (*D*) PR interval, (*E*) QRS interval, (*F*) RR interval, (*G*) QT interval, and (*H*) QTc (Mitchell) using radio-implanted telemeters in control and AnkBp.E1458G^+/+^ mice around 6 months of age (con, N = 7 and AnkBp.E1458G, N = 10). Data passed Shapiro–Wilk normality tests, and unpaired t-tests were performed (*A*–*G*). Data did not pass Shapiro–Wilk normality test, and nonparametric (Mann–Whitney test) was performed (*H*). AnkB, ankyrin-B.

Conscious 6-month-old AnkBp.E1458G^+/+^ mice exhibited sinus bradycardia at rest (mean heart rate [HR] is 658.7 ± 15.2 bpm in control mice *versus* 590.5 ± 21.58 bpm in AnkBp.E1458G^+/+^ mice; Fig. 3, *A* and *B*; *p* = 0.03). Accordingly, the RR interval was significantly prolonged in the AnkBp.E1458G^+/+^ mice compared to the control littermates (mean RR = 0.0919 ± 0.0024 s in control mice *versus* mean RR in AnkBp.E1458G^+/+^ mice = 0.1035 ± 0.0040 s; Fig. 3*F*; *p* = 0.04). No significant changes were noted in the P wave duration, PR interval, or the QRS duration (Fig. 3, *C*–*E* and Table S2). AnkBp.E1458G^+/+^ mice showed a statistically significant prolongation in the QT interval (mean = 0.0206 ± 0.0007 s in control mice *versus* mean = 0.0231 ± 0.0006 s, *p* = 0.02). However, the corrected QT interval (Mitchell *et al*. (11)) trended toward prolongation in the AnkBp.E1458G^+/+^ mice but did not reach statistical significance (mean = 0.0216 ± 0.0006 s in control mice *versus* mean = 0.0228 ± 0.0007 s, *p* = 0.25; Fig. 3, *G* and *H*). There is a debate on whether the QT interval is affected by heart rate in small animals (12, 13, 14), and therefore, we measured both intervals and displayed QTc according to Mitchell *et al* (11).

**Figure 4:**
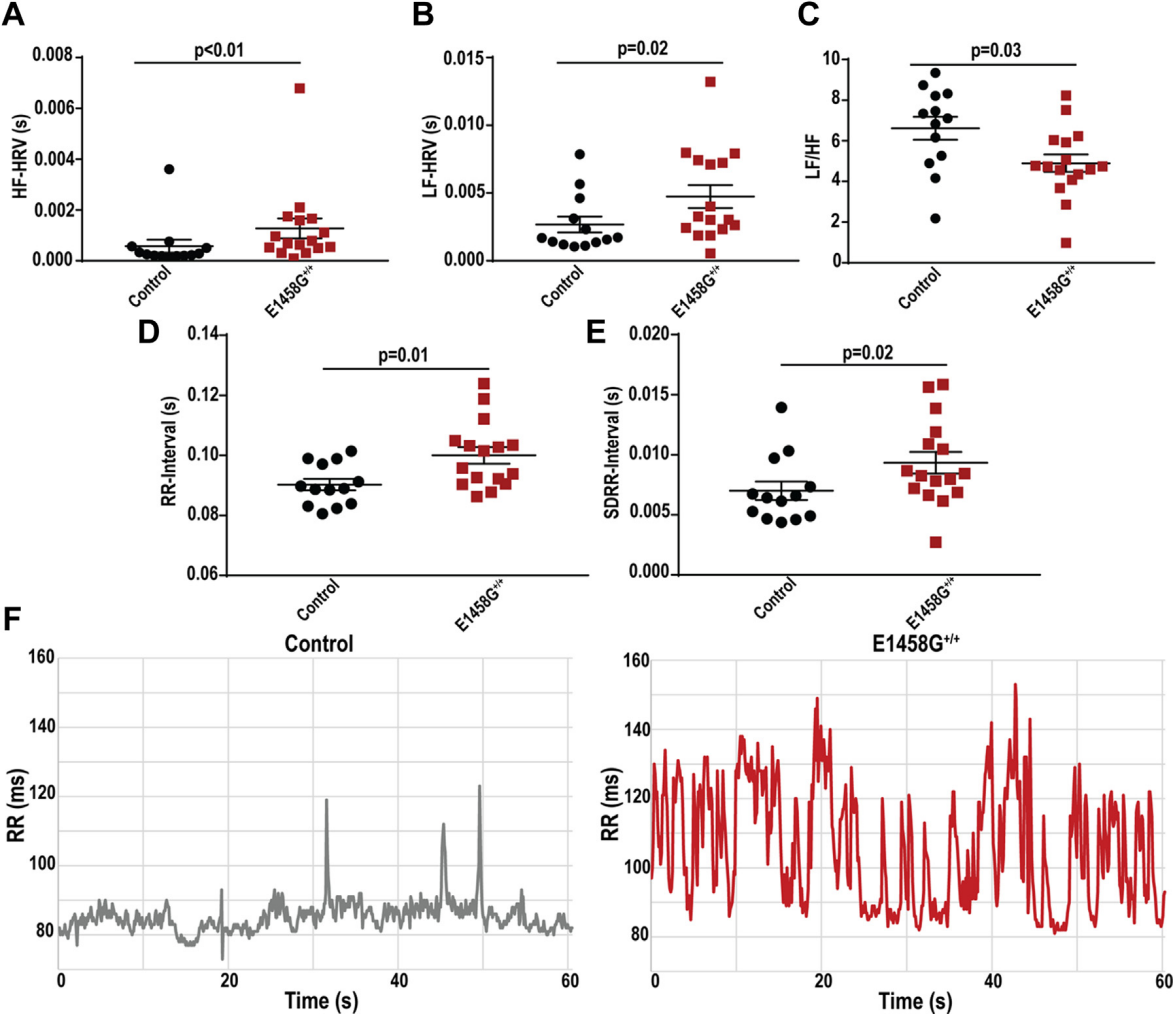
**Aged AnkBp.E1458G**^**+/+**^**mice display heart rate variability at rest.***A*, high frequency (HF) and (*B*) low frequency (LF) spectral components of heart rate variability, HRV. Data did not pass Shapiro–Wilk normality tests, and nonparametric (Mann–Whitney tests) were performed. *C*, LF/HF using Fast Fourier transform (FFT) analysis and radio-implanted telemeters in control and AnkBp.E1458G^+/+^ mice around 6 months of age (con, N = 13 and AnkBp.E1458G^+/+^, N = 16). Data passed Shapiro–Wilk normality test, and unpaired *t* test was performed. *D*, RR interval; data passed Shapiro–Wilk normality test, and unpaired *t* test was performed. *E*, SDRR interval; data did not pass Shapiro–Wilk normality test, and nonparametric (Mann–Whitney test) was performed. *F*, representative traces from control and AnkBp.E1458G^+/+^ mice over 1 min interval. AnkB, ankyrin-B; SDRR, standard deviation of RR.

Abnormal high frequency (HF) and low frequency (LF) components of heart rate variability (HRV) may suggest a dysfunction in autonomic regulation or an intrinsic sinus node dysfunction. Specifically, the HF component is modulated by the parasympathetic nervous system, while the LF reflects the activity of both parasympathetic and sympathetic nervous system (15). The HF and LF spectral components of HRV were significantly increased in the AnkBp.E1458G^+/+^ mice *versus* control littermates at rest (Fig. 4*A* (*p* < 0.01) and B (*p* = 0.02)) in line with mild bradycardia observed in AnkBp.E1458G^+/+^ mice. Notably, LF/HF ratio was significantly reduced in the AnkBp.E1458G^+/+^ mice (Fig. 4*C* *p* = 0.03). Analyzing the RR interval of the control mice *versus* the AnkBp.E1458G^+/+^ mice over a 14 min interval confirmed and displayed both prolongation and variability in the aged mice harboring the single amino acid substitution, AnkBp.E1458G. Analysis of RR and standard deviation of RR intervals displayed a significant increase in both parameters in the AnkBp.E1458G^+/+^ *versus* the control mice (Fig. 4, *D* and *E*). Representative traces from both genotypes over 1 min interval are displayed in Figure 4*F*.

### AnkBp.E1458G^+/+^ mice exhibit arrhythmia in response to adrenergic challenge

**Figure 5:**
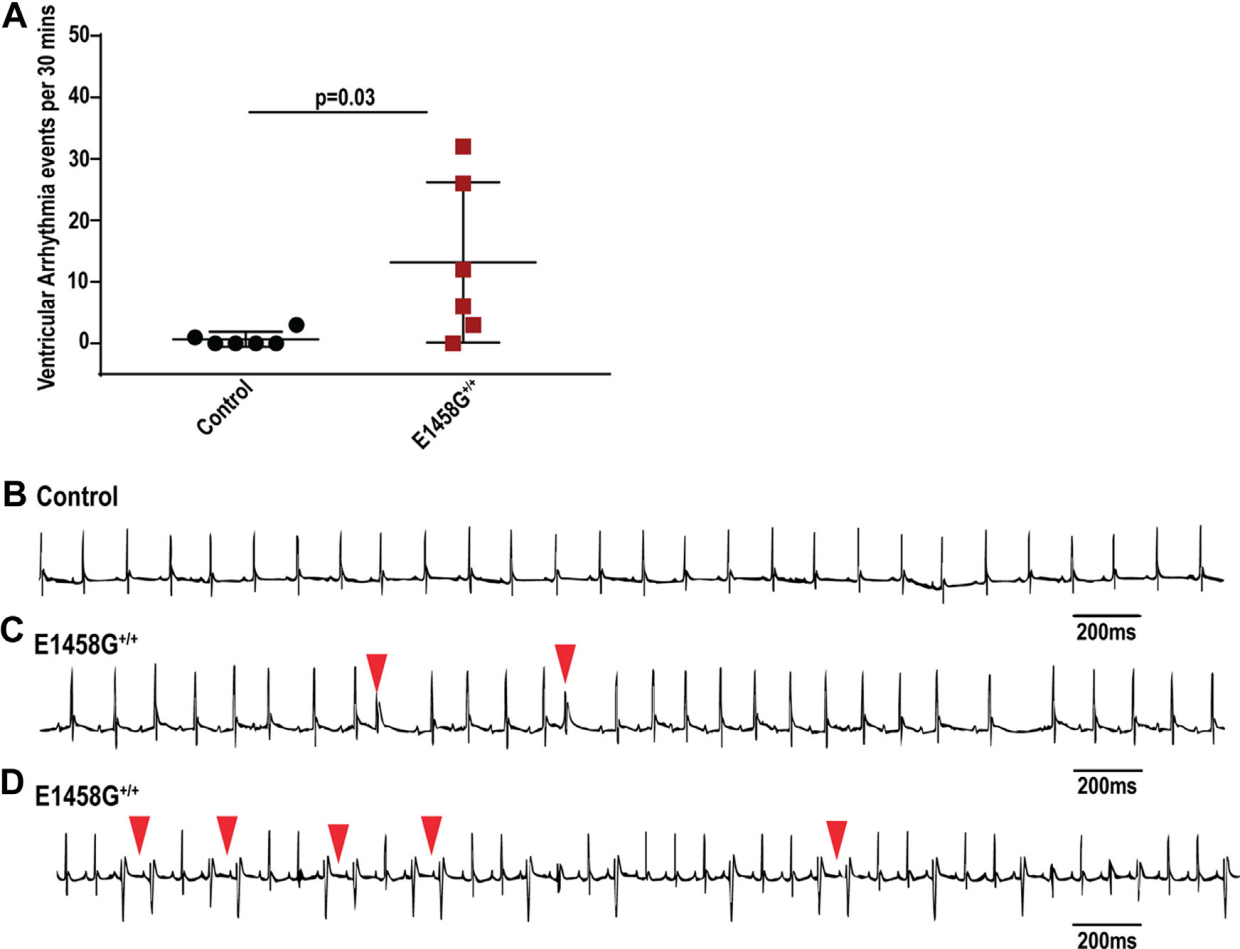
**AnkBp.E1458G**^**+/+**^**mice display arrhythmias following adrenergic stimulation/stress.***A*, AnkBp.E1458G^+/+^ mice display ventricular arrhythmic events after epinephrine stimulation at an early age (∼3 months of age) under isoflurane sedation (con, N = 6 and AnkBp.E1458G^+/+^, N = 6). Data did not pass Shapiro–Wilk normality test, and nonparametric (Mann–Whitney test) was performed. *B*–*D*, representative ECG traces denoting PVCs and chain of couplets in the AnkBp.E1458G^+/+^ mice at early age after epinephrine stimulation and under isoflurane sedation. AnkB, ankyrin-B.

**Figure 6:**
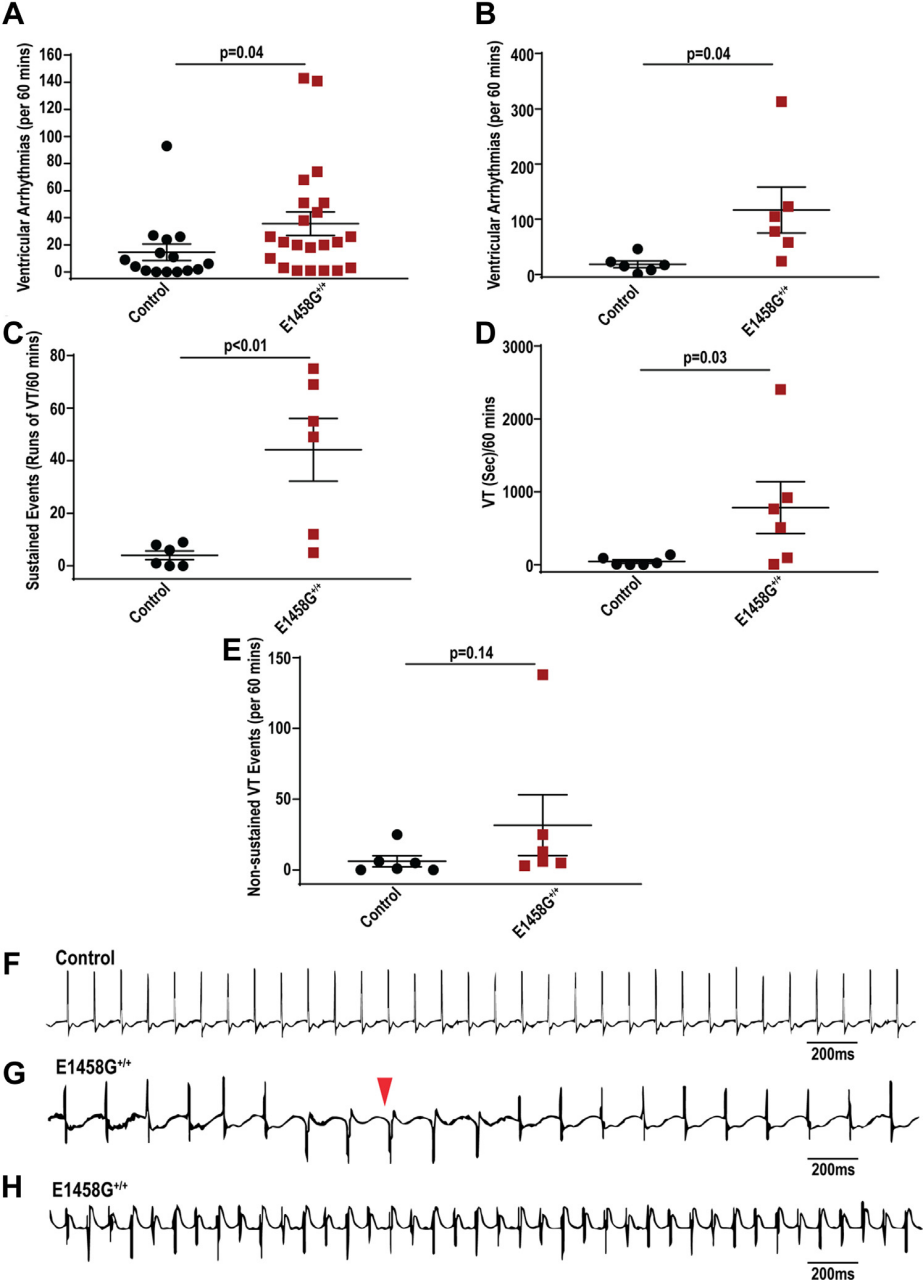
**AnkBp.E1458G**^**+/+**^**mice display a significant pattern of arrhythmia after epinephrine and caffeine stimulation.***A*, AnkBp.E1458G^+/+^ mice show more ventricular arrhythmic events after epinephrine stimulation using implanted telemeters around 6 months of age (con, N = 15 and AnkBp.E1458G^+/+^, N = 22). Data did not pass Shapiro–Wilk normality test, and nonparametric (Mann–Whitney test) was performed. *B*, AnkBp.E1458G^+/+^ mice display significant increase in the number of ventricular arrhythmic events after epinephrine and caffeine stimulation using telemeters around 6 months of age (con, N = 6 and AnkBp.E1458G^+/+^, N = 6). *C*, AnkBp.E1458G^+/+^ mice exhibit an increase in the number of sustained arrhythmic events after epinephrine and caffeine stimulation (con, N = 6 and AnkBp.E1458G^+/+^, N = 6). Data passed Shapiro–Wilk normality tests, and unpaired t-tests were performed (*B* and *C*). *D*, AnkBp.E1458G^+/+^ mice display longer episodes of the sustained events after epinephrine and caffeine stimulation (con, N = 6 and AnkBp.E1458G^+/+^, N = 6). Data did not pass Shapiro–Wilk normality test, and nonparametric (Mann–Whitney test) was performed. *E*, AnkBp.E1458G^+/+^ mice demonstrate a trending increase in number of nonsustained ventricular events (NSVTs) after epinephrine and caffeine stimulation (con, N = 6 and AnkBp.E1458G^+/+^, N = 6). Data did not pass Shapiro–Wilk normality test, and nonparametric (Mann–Whitney test) was performed. *F*–*H*, representative ECG traces denoting nonsustained and sustained arrhythmic events in the AnkBp.E1458G^+/+^ mice after epinephrine and caffeine stimulation. AnkB, ankyrin-B.

Previous studies using *Ank2-*cKO mice noted polymorphic arrhythmia events following adrenergic stimulation (7). Young mice harboring the human variant displayed an increase in the number of ventricular arrhythmic events (control, mean ∼ 0.7 events *versus* AnkBp.E1458G^+/+^ mice, mean ∼18 events) (Fig. 5, *A*–*D*, *p* = 0.0325) when challenged with epinephrine. Moreover, 6-month-old mice also showed a significant increase in the number of ventricular arrhythmia events compared to their control littermates following epinephrine (control, mean ∼14.5 ± 6.14 events and AnkBp.E1458G mice, mean ∼35.8 ± 8.66 events) (Fig. 6*A*, *p* = 0.04). Finally, when challenged with a stress protocol consisting of combined epinephrine (2 mg/kg) and caffeine (120 mg/kg), the 6-month-old AnkBp.E1458G^+/+^ mice displayed a significant increase in the number of ventricular arrhythmic events (control, mean ∼18.3 ± 6.34 events and AnkBp.E1458G mice, mean ∼116.8 ± 41.73 events) (Fig. 6*B*, *p* = 0.04). Notably, the number and duration of sustained episodes of ventricular tachycardia (VT) were significantly higher and prolonged in the AnkBp.E1458G^+/+^ mice compared with control littermates (Fig. 6, *C* and *D*, *p* < 0.01 and *p* = 0.03). The number of nonsustained ventricular tachycardia (NSVT) episodes trended upward in AnkBp.E1458^+/+^ mice (Fig. 6*E*, *p* = 0.14). Representative recordings denoting episodes of VT and NSVT are included in (Fig. 6, *F*–*H*). Taken together, AnkBp.E1458G^+/+^ mice showed arrhythmic events following adrenergic challenge or acute stressor at an early age. These events became more severe and sustained in adult animals at 6 months of age.

**Figure 7:**
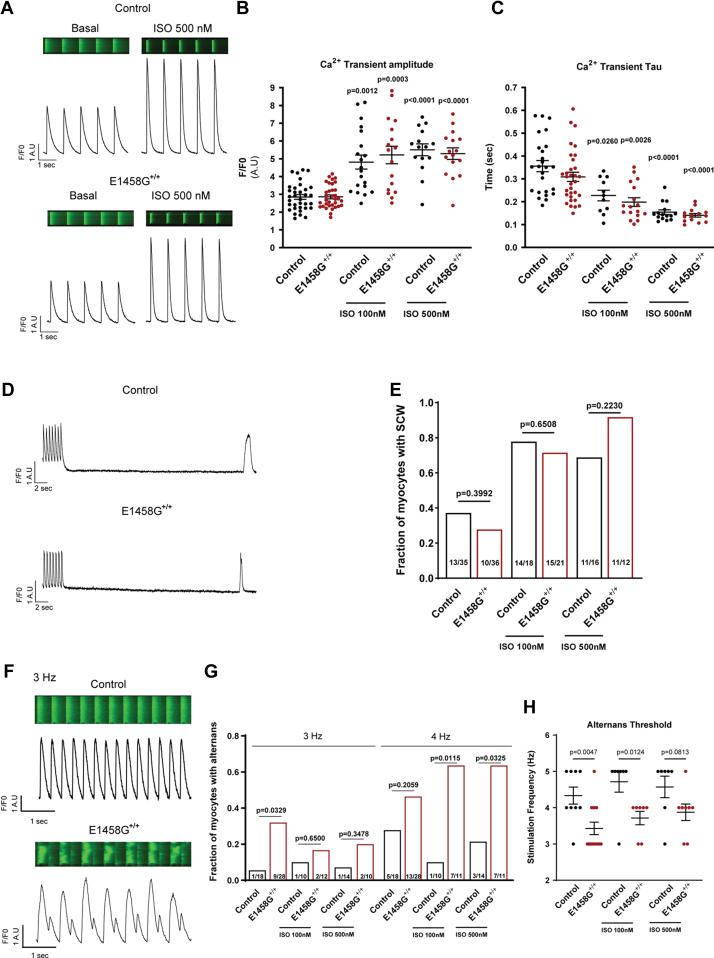
**Calcium handling in AnkBp.E1458G**^**+/+**^**myocytes.***A*, representative confocal line-scan images and Ca^2+^ transient profiles recorded in mouse ventricular myocytes loaded with Fluo-4-AM and stimulated at 1 Hz, with or without 500 nM ISO treatment in control (*upper* panel) and AnkBp.E1458G^+/+^ (*lower* panel) myocytes. *B*, summary data on Ca^2+^ transient amplitude and (*C*) time constant of single exponential decay (Tau) in control and AnkBp.E1458G^+/+^ myocytes with or without ISO treatment at indicated doses. All groups were compared using paired mixed effects ANOVA followed by Tukey correction. *p* < 0.05 *versus* control or AnkBp.E1458G^+/+^ without treatment was considered significant. N = 5 to 7 animals per group (n = 15–34 myocytes). Shapiro–Wilk test was used to evaluate normal distribution. *D*, representative confocal line-scan images of control (*upper* panel) and AnkBp.E1458G^+/+^ myocytes (*lower* panel) showing spontaneous Ca^2+^ waves in 35 s poststimulation at 2 Hz. *E*, comparison of the fraction of myocytes with spontaneous Ca^2+^ waves in 35 s poststimulation at 2 Hz in control and AnkBp.E1458G^+/+^ mice with and without ISO treatment. The presence or absence of spontaneous Ca^2+^ waves was analyzed using a two-tailed Chi-squared test. N = 5 to 7 animals per group (n = 12–36 myocytes). *F*, representative confocal line-scan images of control (*upper* panel) and AnkBp.E1458G^+/+^ myocytes stimulated at 3 Hz, showing the presence of alternans (*lower* panel). *G*, summary data on the fraction of myocytes presenting alternans at 3 Hz or 4 Hz pacing frequency in control and AnkBp.E1458G^+/+^ myocytes with or without ISO treatment. The presence or absence of alternans was analyzed using a two-tailed Chi-squared test. *p* < 0.05 *versus* control in the same condition was considered significant. N = 4 to 5 animals per group (n = 10–28 myocytes). *H*, threshold frequency for the development of alternans. Only myocytes that manifested alternans between 1 to 5 Hz were considered for this analysis. Control and AnkBp.E1458G^+/+^ in each condition were compared by student’s *t* test. *p* < 0.05 *versus* control in the same condition was considered significant. N = 4 to 5 animals per group (n = 7–14 myocytes). AnkB, ankyrin-B; ISO, isoproterenol.

Abnormal calcium handling may lead to cardiac electrical abnormalities either through spontaneous calcium waves that cause delayed afterdepolarizations or through calcium transient alternans that provide a substrate for reentrant mechanisms (16). In this study, we investigated calcium handling in cardiac myocytes derived from AnkBp.E1458G^+/+^ mice and control littermates in the presence (100 nM and 500 nM) and absence of isoproterenol (ISO). As expected, ISO significantly increased the amplitude and rate of decay of calcium transients (Fig. 7, *A*–*C*). We observed that the amplitude and rate of decay of calcium transients were similar between AnkBp.E1458G^+/+^ and control myocytes ± ISO. Additionally, the frequency of spontaneous calcium waves was comparable between the two groups (Fig. 7, *D* and *E*). Notably, AnkBp.E1458G^+/+^ myocytes displayed a higher predisposition to calcium transient alternans than WT myocytes (Fig. 7, *F*–*H*). In line with previous studies (17, 18), ISO tended to decrease the likelihood of alternans in WT myocytes. However, in AnkBp.E1458G^+/+^ myocytes, ISO increased the predisposition to alternans (Fig. 7, *G* and *H*). Overall, these findings illustrate calcium handling dysfunction in AnkBp.E1458G^+/+^ myocytes.

### AnkBp.E1458G^+/+^ mice develop severe cardiac phenotypes in response to chronic cardiac pressure overload

**Figure 8:**
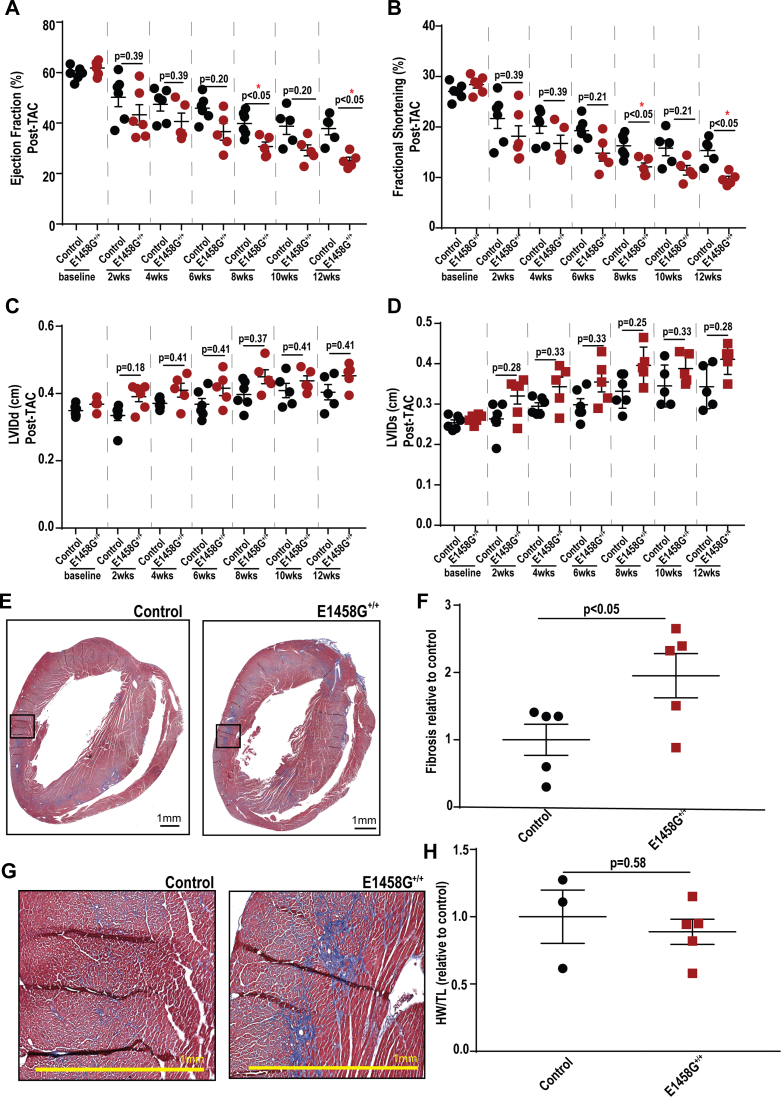
**AnkBp.E1458G**^**+/+**^**mice display severe structural phenotype following pressure overload.***A*, AnkBp.E1458G^+/+^ mice display a reduction in the ejection fraction at 8 and 12 weeks compared to control littermates post TAC surgery (con and AnkBp.E1458G^+/+^, N = 5–6). Repeated measures two-way ANOVA mixed effects model was performed (time effect: *p* < 0.0001, F = 69.98; genotype effect: *p* < 0.05, F = 6.52; time-genotype interaction effect: *p* < 0.01, F = 3.45) with Holm-Šídák multiple comparisons test represented in the figure. *B*, AnkBp.E1458G^+/+^ mice display a reduction in fractional shortening at 8 and 12 weeks compared to control littermates post TAC surgery (con and AnkBp.E1458G^+/+^, N = 5–6). Repeated measures two-way ANOVA mixed effects model was performed (time effect: *p* < 0.0001; F = 73.01, genotype effect: *p* < 0.05, F = 5.74; time-genotype interaction effect: *p* < 0.01, F = 3.23) with Holm-Šídák multiple comparisons test represented in the figure. *C* and *D*, AnkBp.E1458G^+/+^ mice do not display significant changes in left ventricular internal diameter (LVID) during systole and diastole post TAC surgery compared to control littermates. Repeated measures two-way ANOVA mixed effects model was performed ((C) LVIDd, time effect: *p* < 0.0001, F = 12.04; genotype effect: *p* < 0.05, F = 6.94; time-genotype interaction effect: *p* = 0.66, F = 0.68, (D) LVIDs, time effect: *p* < 0.0001, F = 27.28; genotype effect: *p* < 0.05, F = 7.37; time-genotype interaction effect: *p* = 0.22, F = 1.44) with Holm-Šídák multiple comparisons test represented in the figure. *E*–*G*, Masson’s trichrome staining and quantitative analysis in the AnkBp.E1458G^+/+^ mice 12-weeks post TAC surgery (con and AnkBp.E1458G^+/+^, N = 5). Data passed Shapiro–Wilk normality test, and unpaired *t* test was performed. Scale bars represent 1 mm. *H*, HW/TL relative to control post TAC (con, N = 3 and AnkBp.E1458G^+/+^, N = 5). Data passed Shapiro–Wilk normality test, and unpaired *t* test was performed. Mice were around 3 months at baseline before the surgery. AnkB, ankyrin-B; TAC, transverse aortic constriction.

We hypothesized that young AnkBp.E1458G homozygous mice would develop an accelerated structural phenotype in response to chronic cardiac stress. Therefore, a model of pressure overload, transverse aortic constriction (TAC), was performed in young AnkBp.E1458G^+/+^ mice. AnkBp.E1458G^+/+^ mice exhibited a significant reduction in contractility or ejection fraction and fractional shortening at 8 and 12 weeks post TAC compared to control littermates (Fig. 8, *A* and *B*). Moreover, AnkBp.E1458G^+/+^ mice did not display significant differences in the LVID during diastole or systole post TAC (Fig. 8, *C* and *D*). Furthermore, AnkBp.E1458G^+/+^ hearts displayed widespread cardiac fibrosis in the homozygous hearts post TAC (Fig. 8, *E* and *F*) compared with control mice. Comparing the relative expression of cardiac remodeling markers between the young mice (∼2-3 months of age) and mice post TAC, no significant changes were noted in *Myh7*. We observed a significant increase in *Nppa* relative expression in the control and AnkBp.E1458G^+/+^ hearts post TAC *versus* young control hearts (*p* = 0.026 and *p* = 0.025) and *versus* young AnkBp.E1458G^+/+^ hearts (*p* = 0.032 and *p* = 0.0036). *Nppb* relative expression was upregulated in the AnkBp.E1458G^+/+^ hearts post TAC compared to the young controls (*p* = 0.0168) and young AnkBp.E1458G^+/+^ hearts (*p* = 0.0125). *Col1a1* relative expression was upregulated in the AnkBp.E1458G^+/+^ hearts post TAC compared to the young AnkBp.E1458G^+/+^ (*p* = 0.0467). Finally, *Timp1* was significantly upregulated in the control (*p* = 0.0382) and AnkBp.E1458G^+/+^ (*p* = 0.0199) hearts post TAC compared to young AnkBp.E1458G^+/+^ mice (Fig. S4).

### Expression and localization of AnkB membrane partners is altered in AnkBp.E1458G^+/+^ hearts

**Figure 9:**
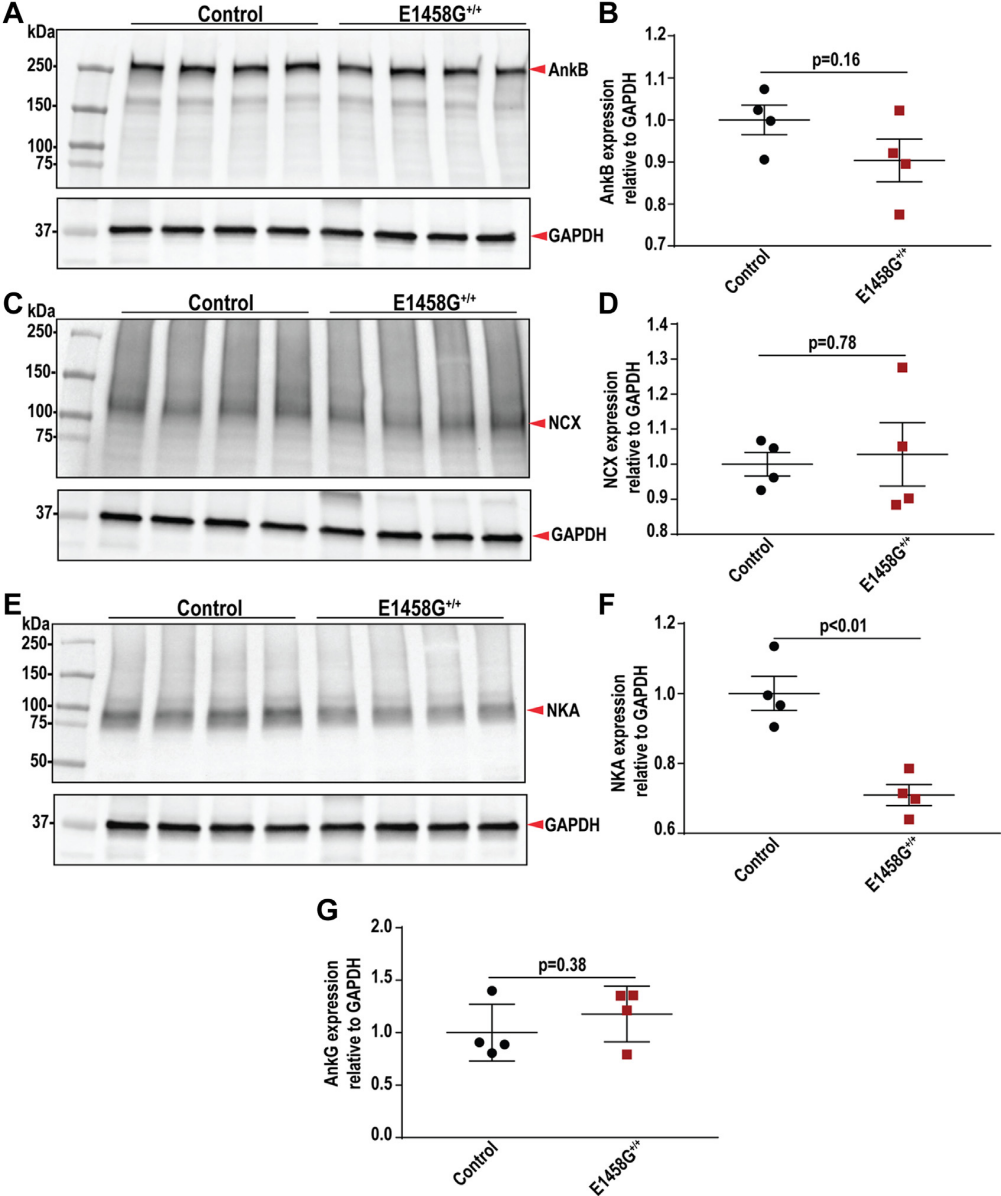
**Expression of ankyrin-B and ankyrin-binding partners in the AnkBp.E1458G**^**+/+**^**heart lysates of aged mice.***A* and *B*, immunoblotting and quantitative analysis of AnkB normalized to GAPDH illustrating no changes in the AnkBp.E1458G^+/+^ hearts. *C* and *D*, immunoblotting and quantitative analysis of Na^+^/Ca^2+^ exchanger (NCX) normalized to GAPDH, showing no changes in the AnkBp.E1458G^+/+^ hearts. *E* and *F*, immunoblotting and quantitative analysis of Na^+^, K^+^-ATPase (NKA) normalized to GAPDH, illustrating a significant reduction in the AnkBp.E1458G^+/+^ hearts around 6 months of age. *G*, no significant changes in the expression of AnkG normalized to GAPDH in the AnkBp.E1458G^+/+^ hearts (con and AnkBp.E1458G^+/+^, N = 4). All data passed Shapiro–Wilk normality tests, and unpaired t-tests were performed. AnkB, ankyrin-B.

We investigated the expression of AnkB and AnkB-key binding partners, including NCX and NKA, in our AnkBp.E1458G^+/+^ mouse model at baseline, older age, and post TAC. Immunoblotting experiments showed no changes in the normalized expression of AnkB in the AnkBp.E1458G^+/+^ mice at 6 months of age (Fig. 9, *A* and *B*). Additionally, *Ank2* mRNA levels were not different between the two groups of mice at ∼6 months of age (Fig. S5). The expression of both NCX and NKA was not different in the young AnkBp.E1458G^+/+^ mice at ∼2 months of age (Fig. S6, *A*–*D*). The expression of NCX was not different between the groups (Fig. 9, *C* and *D*) at ∼6 months of age. However, NKA protein expression normalized to GAPDH showed a significant decrease in the hearts of the aged AnkBp.E1458G^+/+^ mice (∼30% reduction, *p* = 0.0023) (Fig. 9, *E* and *F*). Ankyrin-G expression was unchanged between the two groups of mice (Figs. 9*G* and S7). Notably, NCX expression remained unchanged, and the NKA expression was significantly reduced in the AnkBp.E1458G^+/+^ mice post TAC compared to the control mice (Fig. S6, *E*–*H*). Finally, NKA protein was bound to the AnkB Ig in the presence of the human variant (Fig. S8).

**Figure 10:**
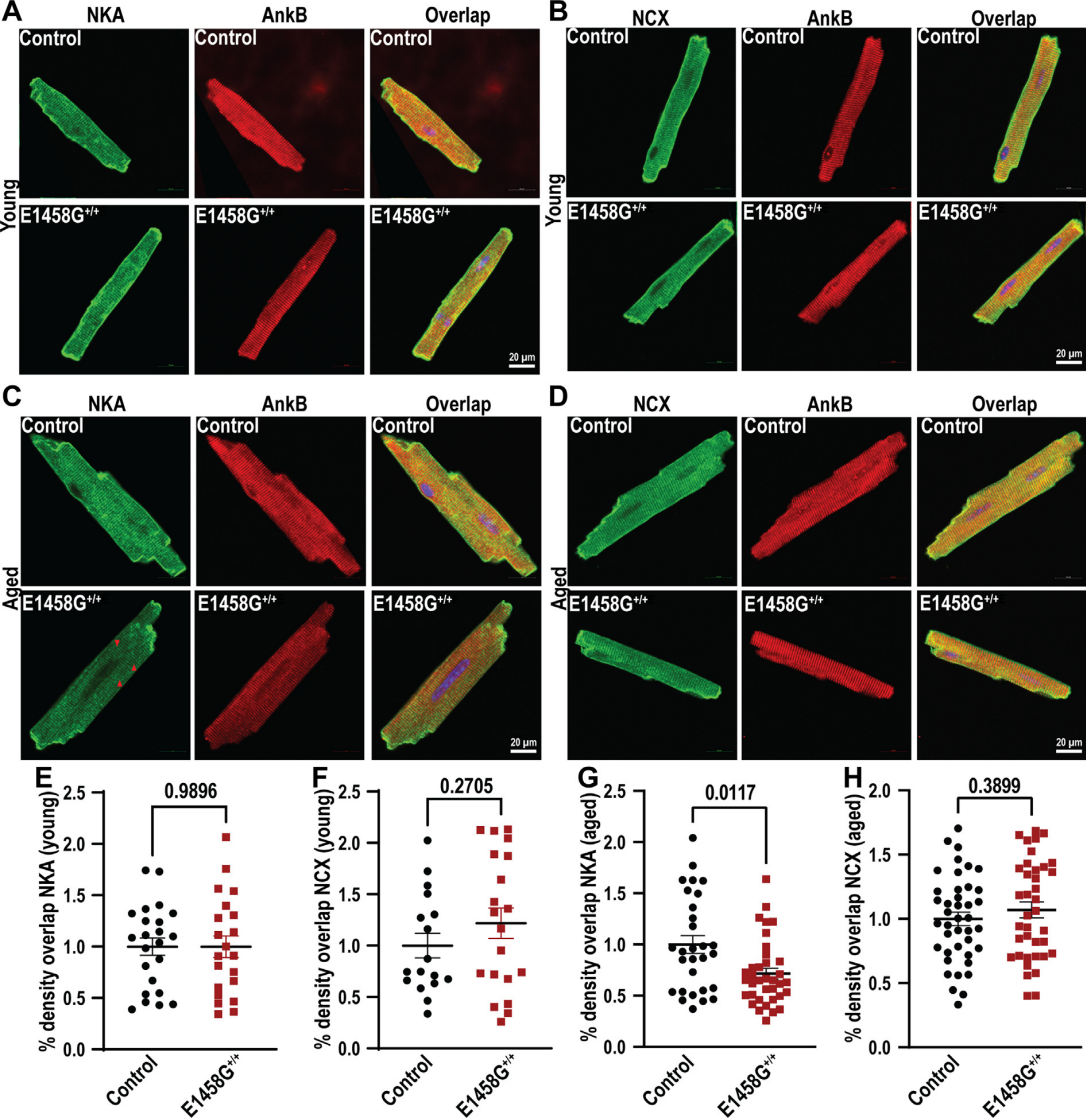
**Expression and localization of AnkB and AnkB membrane partners in the control and AnkBp.E1458G**^**+/+**^**ventricular myocytes.***A*, Na^+^, K^+^-ATPase (NKA) is localized similarly in the ventricular myocytes isolated from the AnkBp.E1458G^+/+^ hearts and control hearts at ∼2 to 3 months of age (con, N = 3, n = 23; and AnkBp.E1458G^+/+^, N = 3, n = 21). *B*, sodium calcium exchanger (NCX) is localized similarly in the AnkBp.E1458G^+/+^ and control ventricular myocytes at ∼2 to 3 months of age (con, N = 3, n = 17; and AnkBp.E1458G^+/+^, N = 3, n = 20). *C*, NKA localization is diminished in AnkBp.E1458G^+/+^ ventricular myocytes in reference to AnkB at ∼6 months of age (con, N = 4, n = 29; and AnkBp.E1458G^+/+^, N = 5, n = 36). *D*, NCX localization remains similar at ∼6 months of age in AnkBp.E1458G^+/+^ and control ventricular myocytes (Con, N = 5, n = 41 and AnkBp.E1458G^+/+^, N = 5, n = 38). Quantitative analysis of immunofluorescence density overlap normalized to control (*E*) NKA in reference to AnkB and (*F*) NCX in reference to AnkB in ∼2 to 3 months-of-age mice. Quantitative analysis of immunofluorescence density overlap normalized to control (*G*) NKA in reference to AnkB and (H) NCX in reference to AnkB in ∼6 months-of-age mice. Data passed Shapiro–Wilk normality tests, and unpaired t-tests were performed (*E*, *F*, and *H*). Data did not pass Shapiro–Wilk normality tests, and nonparametric (Mann–Whitney test) was performed (*G*). Scale bars represent 20 μm. AnkB, ankyrin-B.

AnkB is a cytoskeletal protein that anchors and regulates the organization and localization of both NKA and NCX in a macromolecular complex. We observed no significant changes in the localization of NKA and NCX in AnkBp.E1458G^+/+^ isolated myocytes in relation to AnkB in young mice (Fig. 10, *A*, *B*, *E* and *F*). However, immunolabeling data in the aged AnkBp.E1458G^+/+^ myocytes demonstrated a reduction in NKA localization at the T-tubules (Fig. 10, *C* and *G*). NCX signal was consistently similar in the aged AnkBp.E1458G^+/+^ and control myocytes (Fig. 10, *D* and *H*). In summary, 6-months-old AnkBp.E1458G^+/+^ myocytes displayed altered NKA expression and localization.

## Discussion

The human AnkBp.E1458G variant was initially identified in a large French family with a complex cardiac phenotype, including sinus node dysfunction, ventricular arrhythmia, sudden cardiac death, and atrial fibrillation (6). This phenotype, referred to as “AnkB-syndrome”, has been attributed to altered Ca^2+^ signaling when studied in a full-body haploinsufficient AnkB murine model (7, 19). The AnkBp.E1458G variant has also been implicated in other cardiac conditions, such as ACM (5) and as a potential modifier of wall thickness in hypertrophic cardiomyopathy (7). Notably, the same AnkBp.E1458G variant was identified by Hertz *et al*. in children who suffered from sudden unexpected infant death (20, 21). In summary, the range of cardiac phenotypes identified to harbor the human AnkBp.E1458G variant is diverse and complex.

Despite numerous reports identifying the AnkBp.E1458G missense variant in cases of cardiac disease, the variant has also been identified in nondiseased populations, raising questions about its pathogenicity and penetrance (9, 22). Interestingly, Ware et al. reported that 49 of 64 published *ANK2* variants were either “benign” or “probably benign” (23). AnkB plays a role in the transport and localization of numerous proteins to the membrane, transverse-tubule, and intercalated disc in cardiomyocytes. Although, the AnkBp.E1458G variant is not located where AnkB associates with key binding partners, the variant is localized close to the site critical for AnkB intramolecular association.

GnomAD data estimates the minor allele frequency of the AnkBp.E1458G AnkB variant to be 0.000535 (https://gnomad.broadinstitute.org/variant/4-114269433-A-G?dataset=gnomad_r2_1). Further, the AnkBp.E1458G variant is significantly more prevalent among individuals with European and Latin ancestry. Therefore, it is more frequent in the general population than the AnkB syndrome phenotype (24), consistent with the incomplete penetrance of the AnkB phenotype associated with the AnkBp.E1458G variant. These findings, coupled with the AnkBp.E1458G variant most often being identified in sporadic cases of cardiac disease without clear evidence of familial genotype-phenotype segregation, indicate that the variant must rely on additional genetic and/or environmental factors in order to manifest with a clinical phenotype.

The translational relevance of the haploinsufficient and KO mice has previously been questioned given the strong mouse phenotype compared to the commonly mild human phenotype, coupled with *ANK2* variants implicated in human disease most often being missense (25). Recently, developments in gene editing technology have allowed us to study the specific AnkBp.E1458G variant in mice that may more effectively model the human phenotype. The newly developed KI murine model homozygous for the AnkBp.E1458G variant (Fig. 1) provides a new tool and new insights into the pathogenesis and penetrance of the AnkB syndrome.

Notably, aged AnkBp.E1458G^+/+^ mice displayed bradycardia (Fig. 3) and increased HF and LF spectral elements suggesting higher HR variability in mice at a later age while the mice displayed a lower LF/HF ratio (Fig. 4). Increase in HF and LF is in line with lower basal heart rate observed in AnkBp.E1458G mice. The increase in HRV seen in the KI mice might suggest an intrinsic sinus node dysfunction associated with this variant that leads to increased heart variability as suggested by a previous report (26). However, we cannot exclude that AnkBp.E1458G may also present with increased activity of the parasympathetic nervous system. Additionally, homozygous mice showed increased vulnerability to arrhythmic events in response to adrenergic challenge as early as ∼3 months of age post adrenergic stimulation (Fig. 5). Aged mice showed propensity for more severe cardiac arrhythmic events, more frequent events, and longer sustained runs of arrhythmic episodes in response to adrenergic and caffeine administration (Fig. 6). AnkBp.E1458G^+/+^ myocytes displayed a higher predisposition to calcium transient alternans that provides a potential substrate for reentrant mechanisms (Fig. 7). TAC surgery, a model for chronic cardiac stress, also induced an accelerated heart failure phenotype in the young AnkBp.E1458G^+/+^ mice (Fig. 8). Overall, stress-inducing studies support our hypothesis that the AnkBp.E1458G variant may be more likely to manifest with a cardiac phenotype in response to environmental stressors and aging. Similar results were reported in a truncated Plakophilin-2 mouse model (p.R735X). Plakophilin 2 variants are the most common ACM-causing genetic variants, accounting for 23 to 58% of all ACM cases. However, the ACM phenotype did not manifest in the PKP2p.R735X transgenic mice until exposure to 8 weeks of endurance exercise training (another environmental stressor) (27, 28).

Previous studies have shown NKA and AnkB dissociation as a prominent mechanism for myocardial cell death after myocardial ischemia (29). Calpain-mediated degradation of the NKA anchorage to the membrane cytoskeleton resulted in Na^+^ overload, which had a secondary effect on NCX resulting in secondary Ca^2+^ overload within the cell following ischemia (29). Moreover, Skogestad *et al*. recently demonstrated how disruption in the AnkB–NKA interaction resulted in increased Ca^2+^ sparks (30). Inhibition of NKA raises [Na^+^]_i_ and therefore influences the function of NCX. In order to extrude excess Na^+^, [Ca^2+^]_i_ becomes elevated, which can result in altered Ca^2+^ cycling. Interestingly, Li *et al*. identified that inhibition of NKA during stressed conditions can elicit pro-arrhythmic alternans in guinea pig cardiomyocytes (31). Increased [Ca^2+^]_i_ may result in calcium alternans that may translate into conduction block and reentrant arrhythmia *via* nonuniform refractoriness throughout the electrical signaling pathways (32).

There are limitations to our work. Affected individuals in the setting of AnkBp.E1458G have been in the heterozygous state. Notably, the phenotype of the heterozygous mouse models (usually haploinsufficient) for desmosomal variants are modest (often nothing is observed) and hence desmosomal genetic culprits are also frequently studied in the homozygous state (33). Although gene dosage may be higher, this mouse model still serves as an effective model for evaluating the potential pathologies associated with this AnkB variant. A pressure overload model has not previously been shown to predispose to ACM, and to our knowledge, there is no clinical data to suggest that pressure overload or hypertension exacerbates clinical phenotype in the setting of an *ANK2* variant. Conclusions from this study should be extrapolated with caution to other pathogenic human *ANK2* variants. Gene delivery and pharmacological therapeutics can both be tested *in vivo* using this KI model. While our studies were performed *in vivo*, it will be still crucial for expansion of these studies into large animal models. Human induced pluripotent stem cells would best allow for specific excitation-contraction coupling and further Ca^2+^ regulation studies. In summary, while *ANK2* variants may be loss of function in *in vitro* or even in animal models, our findings support that secondary genetic or environmental factors should be carefully assessed in the context of an ultimate human clinical phenotype.

## Experimental procedures

### Mice

A KI mouse to mimic the human variant at amino acid 1458 of human *ANK2* was generated by The University of Michigan Transgenic Animal Model Core. A single guide RNA was designed to create a CRISPR-induced indel in a region close to codon 1371 in mouse (which corresponds to 1458 in human sequence, ENSMUST00000182078.9). The guide RNA along with Cas9 were microinjected into C57BL/6J and SJL/J zygotes. Chimera mice were screened for the single amino acid substitution at the guide sequence. Germline mice were generated by breeding chimeras to C57BL6/J mice. After germline transmission, mice were genotyped and sequenced to confirm the substitution of the glutamic acid amino acid (E) for glycine (G) (forward: TGTAGACCAGTCCACCAGACACATT; reverse: CCCTGTCTAATTTCTCTAAAGTCAGAGGC). Mice were backcrossed on a C57BL/6 background for five generations. The whole colony was produced from a single founder. Age-matched male and female mice were used in experiments throughout the manuscript. All animal experiments were conducted in accordance with the Ohio State University Institutional Animal Care and Use Committee guidelines (IACUC Protocol Number 2011A00000034).

### Echocardiography

Transthoracic echocardiography using (GE Logiq e) with the L10-22 (mHz) transducer was performed on mice anesthetized using 2% isoflurane in 1 L/min oxygen. Anesthesia was maintained by administration of oxygen and approximately 1.25% isoflurane during the whole imaging procedure. HR was monitored throughout imaging, and recordings taken at an HR less than 400bpm were excluded from the analysis. Mice were immobilized on a heated imaging stage during image acquisition, and a temperature probe was inserted into the rectum of the mouse to monitor its core temperature of approximately 37 °C. Electrode gel was placed on the ECG sensors of the heated platform. Parasternal long-axis images inclusive of two-dimensional loops and freeze-frame, end-diastolic images were collected to measure end-diastolic LV cavity dimension (LVID, d) and posterior wall thickness (LVPW, d). Parasternal short axis images at the level of papillary muscles using M-mode were also collected to determine LVPW and LVID, in both systole (s) and diastole (d), as previously described (34). Parasternal short axis and M mode measurements were used to calculate functional parameters including fractional shortening (FS%) and ejection fraction (EF%).

### Electrocardiogram

Surface electrocardiogram analysis was conducted on mice anesthetized with isoflurane (2% in 1 L/min oxygen). Anesthesia was maintained by administration of oxygen and approximately 1% isoflurane during the whole recording procedure. Mice were immobilized on a heated imaging stage during acquisition. Lead II ECGs were collected using PowerLab equipment (AD Instruments). Subsurface ECG recordings were collected from anesthetized mice around 3 months of age for a 5-min interval of recording. Mice at ∼3 months of age were stimulated with epinephrine (2 mg/kg) under isoflurane sedation, and ventricular arrhythmic events were calculated over a 30-min period. Conscious ECGs were also collected using implanted radio telemetry devices using an ETA-F10 miniature telemeter (DSI) and Dataquest A.R.T acquisition software (https://www.datasci.com/products/software/ponemah) around 6 months of age for baseline and poststimulation measurements. All conscious ECGs were analyzed using LabChart software (https://www.adinstruments.com/support/software). Researchers blinded to genotype performed data collection and analysis. Conscious mice were stimulated with epinephrine (2 mg/kg). After a week of rest, mice were stimulated with epinephrine (2 mg/kg) and caffeine (120 mg/kg, Millipore Sigma C1778) at the same time. ECG recordings were collected before stimulation to measure baseline ECG features. Mice were then recorded for an hour poststimulation. The total number of ventricular arrhythmic events were calculated by combining the number of arrhythmic events such as bigeminy (>10 consecutive abnormal ventricular beats alternating with normal ventricular beats), VT (>10 consecutive beats of tachycardia), NSVT (three to 10 consecutive beats), and isolated ventricular arrhythmic events such as premature ventricular contractions and ventricular couplets.

### Immunoblotting

Heart tissue samples were isolated from 7-week- and 6-month-old mice after being euthanized using CO_2_ and homogenized using a Cryolys-cooled Precellys 24 bead homogenizer (Bertin Corp.) using a combination of 1.4 mm and 2.8 mm ceramic beads at 6000 rpm for three bouts of 15 s in homogenization buffer (0.025 M Tris–HCl, 0.15 M NaCl, 0.001 M EDTA, 1% v/v NP-40, 5% v/v glycerol, pH=7.4), as previously described (35). Homogenates were then centrifuged for 30 min at high speed at 4 °C. Following quantification by bicinchoninic acid assay (Pierce), 30 to 40 μg of lysates were separated on 4 to 15% precast ProteanTGX gels (Bio-Rad) and transferred to nitrocellulose membranes. Membranes were blocked at room temperature (RT) for 1 h and then incubated in primary antibody overnight at 4 °C. Primary antibodies targeted AnkB (1:1000, Covance antibody) (7), NCX1 (1:1000, Cell Signaling Technology CST 79350), NKA (1:1000, Cell Signaling Technology CST 3010), and Ankyrin-G (1:1000, Covance, a gift from Dr Paul Jenkins) (36). Secondary antibodies used were donkey anti-rabbit or donkey anti-mouse (Jackson ImmunoResearch Laboratories, Bio-Rad). Densitometry analysis was performed using ImageJ software (https://imagej.net/ij/index.html).

### Histologic analysis

Hearts were excised and fixed in 10% neutral buffered formalin for 24 h and then stored in 70% ethanol at 4 °C until sectioning. Masson’s trichrome staining was performed on 5 μm sections at the histology core at the Wexner Medical Center at the Ohio State University. Trichrome-stained cardiac sections were collected on an EVOS microscope (Thermo Fisher Scientific). Researchers blinded to genotype performed image collection and analysis. Whole-heart images were edited in Adobe Photoshop to excise aortic tissue, as the focus of the study is intra-myocardial fibrosis. Fibrosis was quantified using an add-on to MATLAB (Mathworks). The add-on converts images from the RGB color space into CIELAB color space, then segments images using the kmeans algorithm as previously described (35). Finally, the fibrosis (blue) segment is filtered through a color mask to remove noise, and the ratio of fibrosis to healthy red tissue is calculated.

### Isolation of cardiomyocytes

Adult mouse ventricular cardiomyocytes were prepared as previously described (37). Murine hearts from male and female mice were obtained after animals were euthanized by CO2 asphyxiation followed by cervical dislocation in accordance with the Guide for the Care and Use of Laboratory Animals published by the National Institutes of Health and the Ohio State University Institutional Animal Care and Use Committee-approved protocols. Following isolation, cells were fixed in ethanol and stored at −20 °C till staining experiments.

### Immunofluorescence

Adult ventricular myocytes were isolated and processed for immunofluorescence as previously described (38). For adult ventricular myocytes, staining experiments were performed in solution. Briefly, cells were blocked for 1 h in blocking solution (3% fish gel, 0.75% triton-100(10%) and 1% DMSO). Cells were incubated in primary antibodies at 4 °C overnight while rotating. Primary antibodies included NCX (Swant R3F1, 1:100), Ank-B (1:100, Covance antibody) (7), and NKA (Abcam ab7671, 1:100). Secondary antibodies, including anti-rabbit and anti-mouse conjugated to Alexa-Fluor, were applied to the samples for 2 h at RT. For control experiments, parallel samples were incubated with secondary antibodies and with primary antibody controls for 2 h at RT and processed to ensure lack of nonspecific secondary antibody staining. After secondary antibody incubation for 2 h, cells were extensively washed, applied to the slides in Vectashield imaging medium (Vector Laboratories), and then coverslips (#1) were applied. Images were collected on a confocal microscope (LSM 510 Meta, Zeiss). Images were collected using identical confocal protocol settings at RT, and the observer was blinded to the genotype. Quantification of signal intensity was performed using ImageJ software. Area of overlap between two signals was calculated using region of interest manager under ImageJ software. All calculations were normalized to control.

### Detection of cytosolic Ca^2+^ by confocal microscopy

Control or AnkBp.E1458G^+/+^ myocytes were loaded with 10 μM Fluo-4-AM (Thermo Fisher Scientific) for 20 min at RT. After 10 to 20 min of de-esterification in free dye solution, the myocytes were mounted in an imaging chamber (Warner Instruments) and continuously superfused with an external solution containing the following(in mM): 137.0 NaCl, 5.4 KCl, 4.5 MgCl_2_, 0.16 NaH_2_PO_4_, 3 NaHCO_3_, 20.0 Hepes, 10.0 glucose, 10.4 taurine, 1.8 CaCl_2_. Intracellular Ca^2+^ transients were induced by electrical field stimulation (SD9 stimulator, Grass Technologies/Astro-Med Inc). After stabilization (usually 3–5 min), imaging was performed using Olympus FluoView FV 1000 (Olympus America Inc) confocal microscope system equipped with x60 oil-immersion objective lens (NA 1.4). Fluo-4 was excited with a 488 nm line of argon laser, and the signal was collected at 500 to 600 nm wavelengths. Line-scanning (512 × 512 pixels, 2.0 μS/pixel, and 2.1 msec per line) was performed along the longitudinal axis of cells (avoiding nuclei). Fluorescence signals were normalized to the baseline cellular fluorescence (F0). Ca^2+^ transients were analyzed as the F/F0 mean value over 10 to 15 transients for each image. In the experiments with ISO treatment, the drug was administered to the perfusion buffer. Spontaneous Ca^2+^ waves were evaluated during 35 s after stimulation at 2 Hz. Ca^2+^ alternans were induced incrementally by increasing the pacing frequency (1–5 Hz).

### Transverse aortic constriction

Mice were anesthetized with 2% isoflurane and intubated for artificial ventilation at 120 to 160 breaths per minute, tidal volume of 0.2 to 0.35 ml. A heating pad was used to keep body temperatures at 37 °C throughout the procedure. The procedure was performed on 3-month-old mice as previously described (35). Briefly, the transverse aorta was accessed *via* a left lateral thoracotomy and the aorta was ligated overlying a blunted 27-gauge needle. The needle was removed immediately following ligation, leaving a discrete region of stenosis at the aorta. Successful constriction was confirmed by checking the aortic root and the constriction site using echocardiography. The surgeon was blinded to genotype.

### Quantitative real-time PCR

Real-time PCR was performed as previously described (34). Briefly, total RNA from the mouse heart tissues was extracted with TRIzol Reagent (Invitrogen) following manufacturer's instructions; 1 μg of total RNA, treated with ezDNase, was used for the first-strand complementary DNA synthesis using SuperScript IV Vilo Master Mix (Thermo Fisher Scientific). Quantitative reverse transcription polymerase chain reactions were performed in triplicate in 96-well optical plates with PowerUp SYBR Green Master Mix (Thermo Fisher Scientific). Primers for *Ank2-* F: TACAACCAACGTGTCTGCCA and R: TGCAAAGGCAACAGACTCCT; *Nppa*- F: 5′-CTGGACTGGGGAGGTCAAC-3′ and R: 5′-AGGGCAGATCTATCGGAGGG-3′; *Nppb*- F: 5′-GCTCCCCAATCCATCACAGA-3′ and R: 5′-CTGCCTTGAGACCGAAGGAC-3’; *Myh7-* F: 5-TGACAGAGGAGATGGCTGGT-3′ and R: 5′-CCTTGGCCTTGGTCAGAGTA-3′; *Col1a1*- F: 5′-TCCTTCCTCTACACAGGGTCC-3′ and R: 5′-CGGCCACCATCTTGAGACTT-3′; *Timp1*- F: 5′-GGCATCTGGCATCCTCTTGT-3′ and R: 5′-CAGGTCCGAGTTGCAGAAGG-3′. *Hprt* levels were used as a normalization control.

### FFT analysis

Heart rate variability was measured *via* fast Fourier transformation (FFT)-based time- and frequency-domain analysis. Custom MATLAB script (39) allowed FFT analysis of text files with every raw RR interval within a period of time. To prepare data for MATLAB analysis, ECG recordings were edited on LabChart, and all noise was removed. Each RR interval was then measured by LabChart and exported into a text file in 2-min increments to screen for potential noise missed in the initial edit. A total of 14 min was analyzed for RR and standard deviation of RR intervals and also FFT was analyzed to determine LF and HF spectra, LF/HF, etc. (LF range 0.15–1.5 Hz, HF range 1.5–5 Hz).

### Immunoprecipitation

Control and AnkBp.E1458G^+/+^ mouse heart samples were homogenized in buffer (containing 0.025 m Tris–HCl, 0.15 m NaCl, 0.001 m EDTA, 1% (v/v) Nonidet P-40, 5% (v/v) glycerol, pH 7.4) using the bead homogenizer. Lysates were centrifuged for 30 min at 13,000 rpm at 4 °C. Four hundred micrograms of supernatant was incubated and rotated with AnkB-Ig (Covance, custom-made antibody) or control IgG at 4 °C overnight. Following incubation, lysates with antibodies were rotated and incubated with Protein A/G Magnetic Beads (Pierce, number 88802) for 4 to 5 h at 4 °C. The supernatant was then removed from the beads using a magnetic stand, and the beads were washed 3 times with PBS containing 500 mM NaCl. Bound protein was eluted with 2 × Laemmli sample buffer and β-mercaptoethanol and heated to 95 °C for 10 min before immunoblotting with NKA (1:750, Cell Signaling Technology CST 3010).

## Statistics

Data are presented as mean ± SEM. For the comparison of two groups, we performed unpaired two-tailed student *t* test when data passed normality test (Shapiro–Wilk normality test). When data did not pass Shapiro–Wilk normality test, we performed Mann-Whitney U test (nonparametric for unpaired comparison) to compare two groups. For comparison of more than two groups, ANOVA test was performed when data passed Shapiro–Wilk normality test followed by Tukey’s multiple comparisons test. When data did not pass Shapiro–Wilk normality test, Kruskal–Wallis test (nonparametric) was performed followed by Dunn’s multiple comparisons test. For cardiac function parameters (EF, FS, LVIDd, LVIDs) measured over time in response to TAC, data was screened graphically with model residuals, homoskedasticity, and QQ plots to ensure the applicability of a two-way ANOVA. A two-way ANOVA mixed effects model for repeated measures was then used with post hoc Holm-Šídák multiple comparisons test. Sphericity was not assumed, and the Geisser-Greenhouse correction was deployed. For our study, a value of *p* < 0.05 was considered statistically significant.

## Data availability

All data are available within the manuscript or supporting information.

## Supporting information

This article containssupporting information.

## Conflict of interest

The authors declare that they have no conflicts of interest with the contents of this article.
