# Supplementary material for: Impact of stress on cardiac phenotypes in mice harboring an ankyrin-B disease variant
Source: J Biol Chem. 2023 May 12;299(6):104818. doi: 10.1016/j.jbc.2023.104818 (PMC10318515; doi:10.1016/j.jbc.2023.104818)
Supplement: Supporting information [file mmc1.pdf]

Supporting Information

Impact of stress on cardiac phenotypes in mice harboring an ankyrin-B disease variant

Michael J. Wallace [1,2], Nipun Malhotra[1,3], Juan Ignacio Elio Mariángelo[1,2], Tyler L. Stevens[1,2], Lindsay J. Young[1], Steve Antwi-Boasiako[1], Danielle Abdallah[1], Sarah Sumie Takenaka[1], Omer Cavus[1], Nathaniel P. Murphy[1], Mei Han[1], Xianyao Xu[1], Matteo E. Mangoni[4], Thomas J. Hund[1,5,6], Jason D. Roberts[7], Sandor Györke[1,2], Peter J. Mohler[1,2,6] and Mona El Refaey[1,3*]

[1]The Frick Center for Heart Failure and Arrhythmia, Dorothy M. Davis Heart and Lung Research Institute, [2]Department of Physiology and Cell Biology, [3]Department of Surgery/Division of Cardiac Surgery, The Ohio State University, Columbus, OH, USA.

[4]Institut de Génomique Fonctionnelle, Université de Montpellier, CNRS, INSERM, Montpellier, France.

[5]Department of Biomedical Engineering, College of Engineering, The Ohio State University, Columbus, Ohio, USA.

[6]Department of Internal Medicine/Division of Cardiovascular Medicine, The Ohio State University, Columbus, OH, USA.

[7]Population Health Research Institute, McMaster University, and Hamilton Health Sciences, Hamilton, Ontario, Canada.

Running Title: Impact of stress on the penetrance of ankyrin-B syndrome

***Address correspondence to**

Mona El Refaey, PhD, MS

Department of Surgery/Division of Cardiac Surgery

The Dorothy M. Davis Heart and Lung Research Institute

The Ohio State University College of Medicine and Wexner Medical Center

333 West 10th Avenue, Columbus, OH, 43210

Mona.elrefaey@osumc.edu

Tel: 614-366-2748

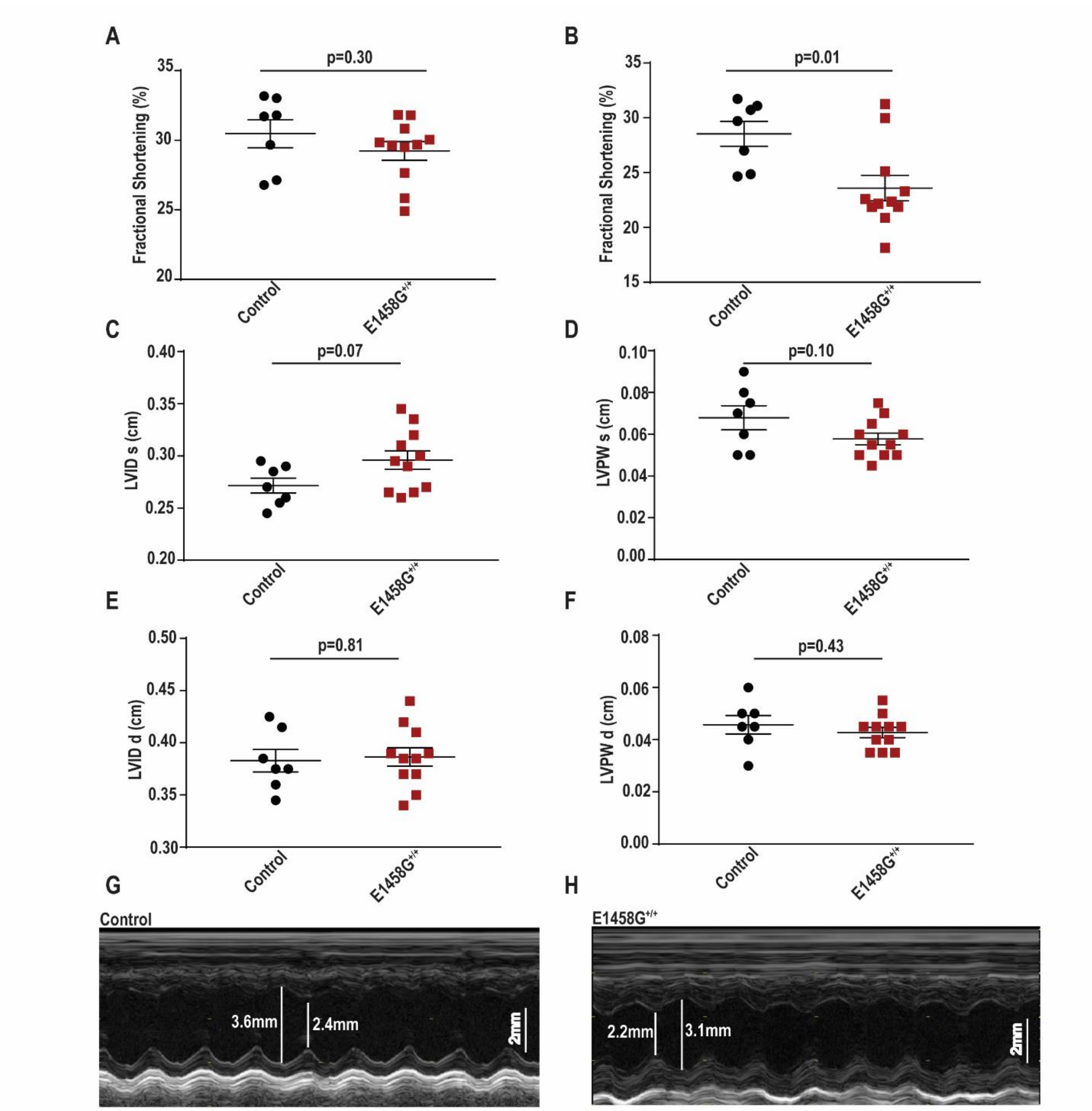

Suppl. Figure 1. AnkBp.E1458G$^{+/+}$ mice do not display an early structural phenotype. A. Control and AnkBp.E1458G$^{+/+}$ mice do not display changes in fractional shortening around three months of age (Con, N=7 and AnkBp.E1458G$^{+/+}$, N=11). Data passed Shapiro-Wilk normality test and unpaired t-test was performed. **B.** AnkBp.E1458G$^{+/+}$ mice display a significant reduction in fractional shortening around six months of age (Con, N=7 and AnkBp.E1458G$^{+/+}$, N=11). Data passed Shapiro-Wilk normality test and unpaired t-test was performed. **C-D** AnkBp.E1458G$^{+/+}$ mice display a trending increase in left ventricle internal diameter (LVID) and a trending decrease in left ventricle posterior wall (LVPW) during systole around six months of age (Con, N=7 and AnkBp.E1458G$^{+/+}$, N=11). Data passed Shapiro-Wilk normality tests and unpaired t-tests were performed. **E-F.** No significant changes in the left ventricle internal diameter (LVID)

and left ventricle posterior wall thickness (LVPW) during diastole around six months of age (Con, N=7 and AnkBp.E1458G$^{+/+}$, N=11). Data passed Shapiro-Wilk normality tests and unpaired t-tests were performed**. G-H.** Representative echocardiographs denoting no changes in cardiac function in the mice carrying the human AnkBp.E1458G variant and the control littermates around three months of age (Con, N=7 and AnkBp.E1458G$^{+/+}$, N=11), Scale bars equal 2mm.

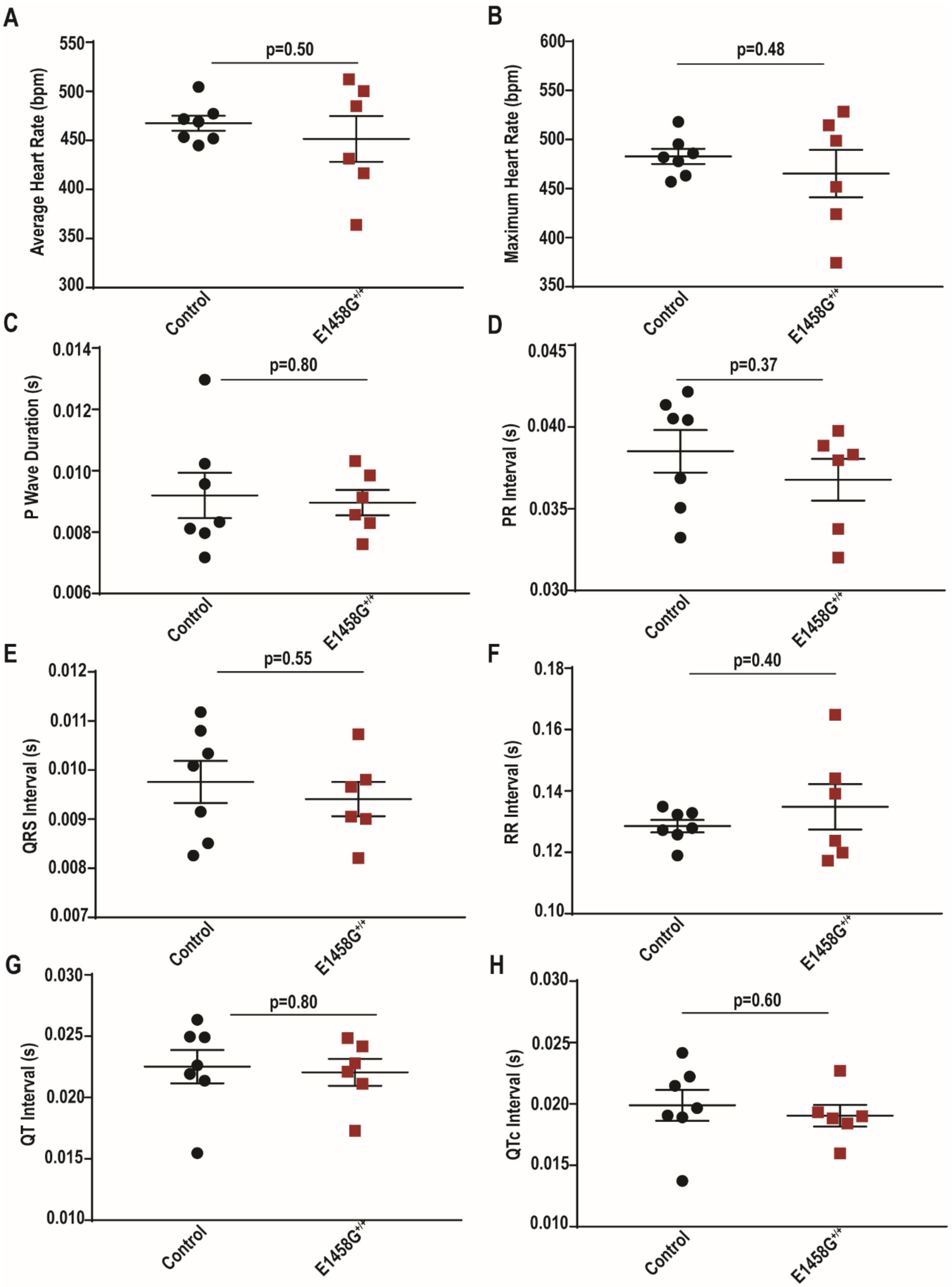

Suppl. Figure 2. AnkBp.E1458G$^{+/+}$ mice do not display electrical changes in subsurface ECG around three months of age. A. Average Heart rate**, B.** Maximal heart

rate, **C.** P Wave Duration, **D.** PR Interval, **E.** QRS Interval, **F.** RR Interval, **G.** QT Interval and **H.** QTc (Mitchell) (Con, N=7 and AnkBp.E1458G$^{+/+}$, N=6). All data passed Shapiro-Wilk normality tests and unpaired t-tests were performed**.**

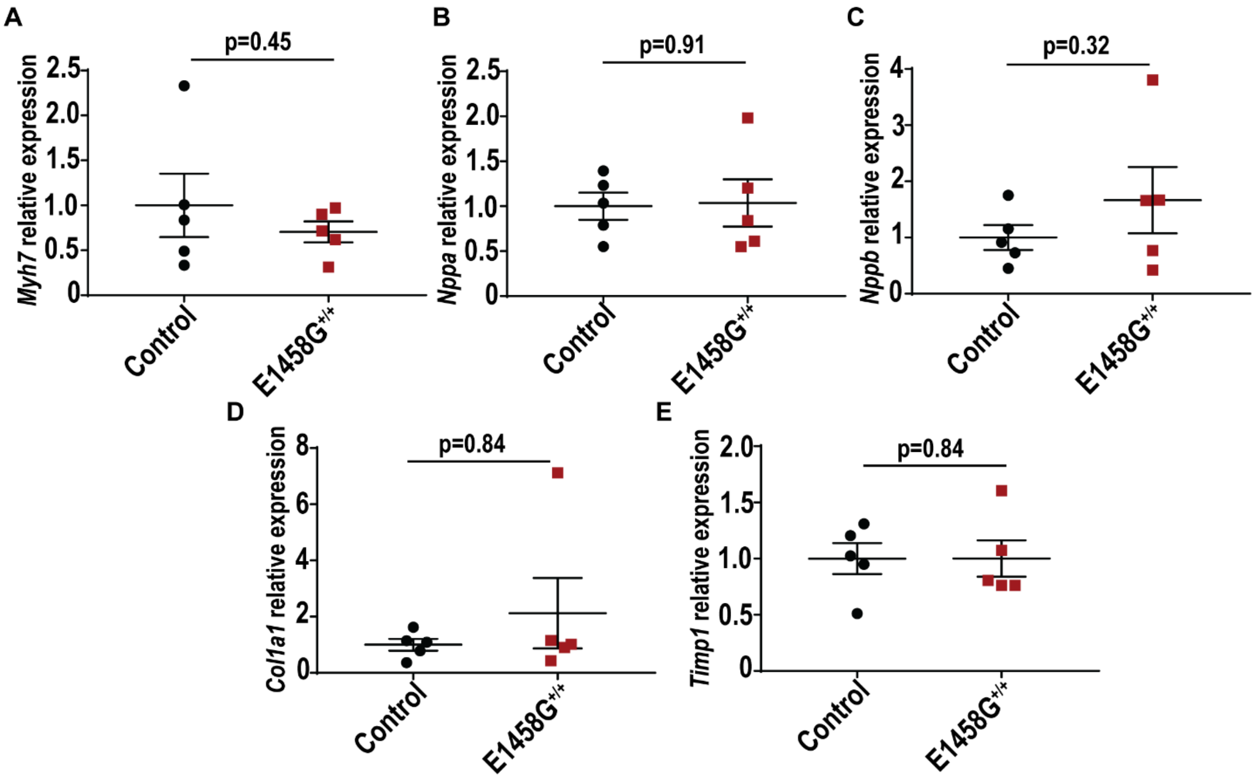

Suppl. Figure 3. No changes in hypertrophic gene expression markers; (A) myosin heavy chain 7 (*Myh7*), (B) atrial natriuretic peptide (*Nppa*), (C) brain natriuretic peptide (*Nppb*), (D) Collagen type I alpha 1 chain (*Col1a1)* and (E) Tissue inhibitor matrix metalloproteinase 1 (*Timp1)* in WT and AnkBp.E1458G$^{+/+}$ mice at ~six months of age (N=5/ genotype). Data passed Shapiro-Wilk normality tests and unpaired t-tests were performed (A-C). Data did not pass Shapiro-Wilk normality tests and Mann-Whitney tests were performed (D-E).

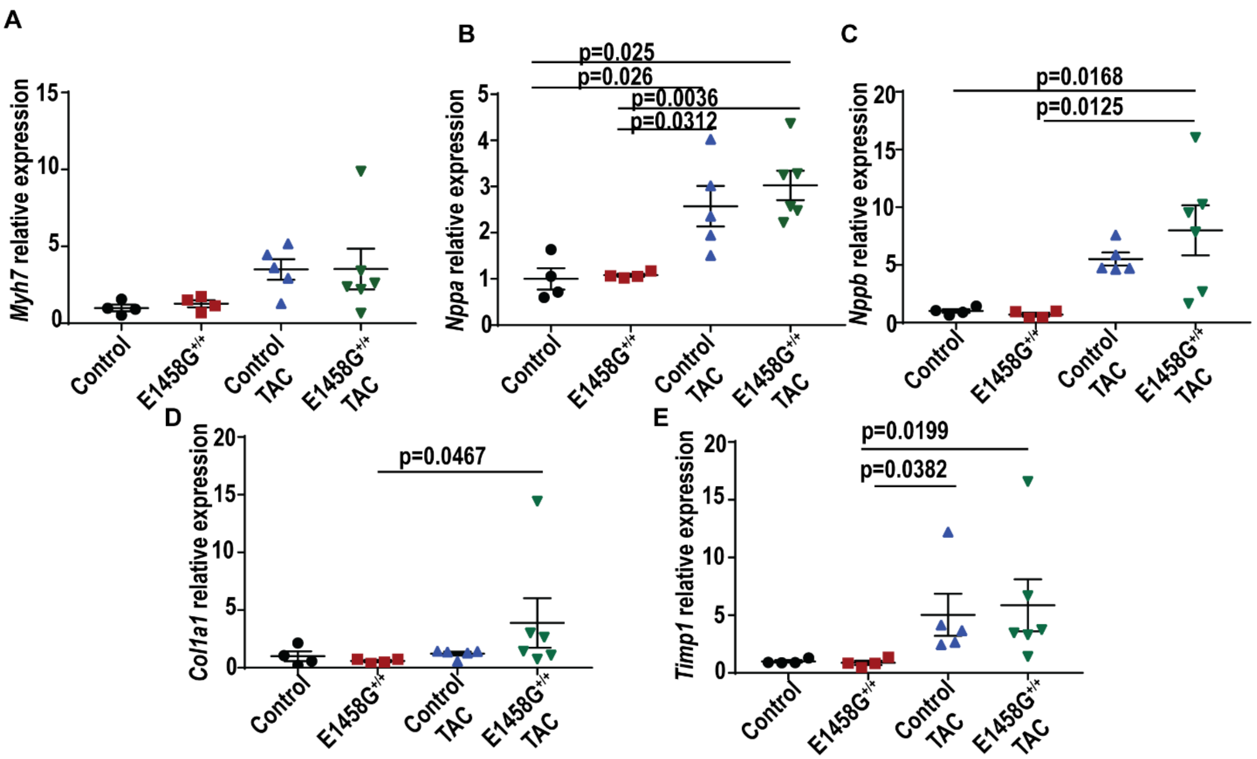

Suppl. Figure 4. Hypertrophic gene expression markers: (A) myosin heavy chain 7 (*Myh7*), data did not pass Shapiro-Wilk normality test and Kruskal-Wallis test was performed followed by Dunn's multiple comparisons test. (B) atrial natriuretic peptide (*Nppa*), data passed Shapiro-Wilk normality test and ANOVA test was performed followed by Tukey's multiple comparisons test. (C) brain natriuretic peptide (*Nppb*), data passed Shapiro-Wilk normality test and ANOVA test was performed followed by Tukey's multiple comparisons test. (D) Collagen type I alpha 1 chain (*Col1a1),* data did not pass Shapiro-Wilk normality test and Kruskal-Wallis test was performed followed by Dunn's multiple comparisons and (E) Tissue inhibitor matrix metalloproteinase 1 (*Timp1)*, data did not pass Shapiro-Wilk normality test and Kruskal-Wallis test was performed followed by Dunn's multiple comparisons test. WT and AnkBp.E1458G$^{+/+}$ hearts were used at baseline (~2-3 months of age) and 4 weeks post TAC (N=4-5/group).

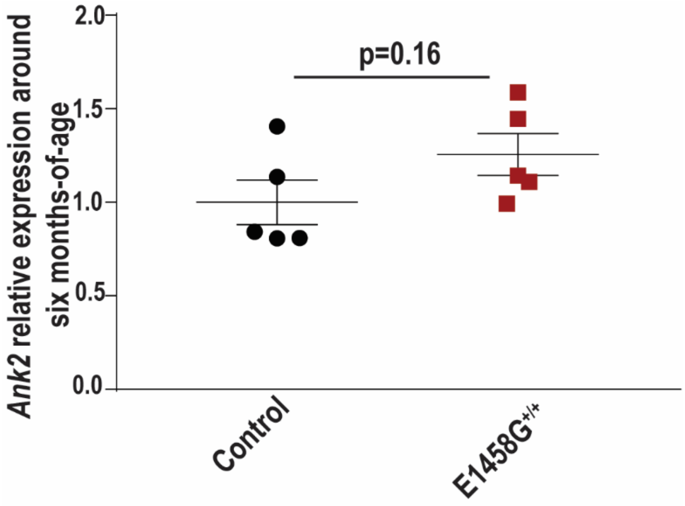

Suppl. Figure 5. *Ank2* relative expression in control and AnkBp.E1458G$^{+/+}$ mice around six months of age (N=5 mice/genotype). Data passed Shapiro-Wilk normality test and unpaired t-test was performed**.**

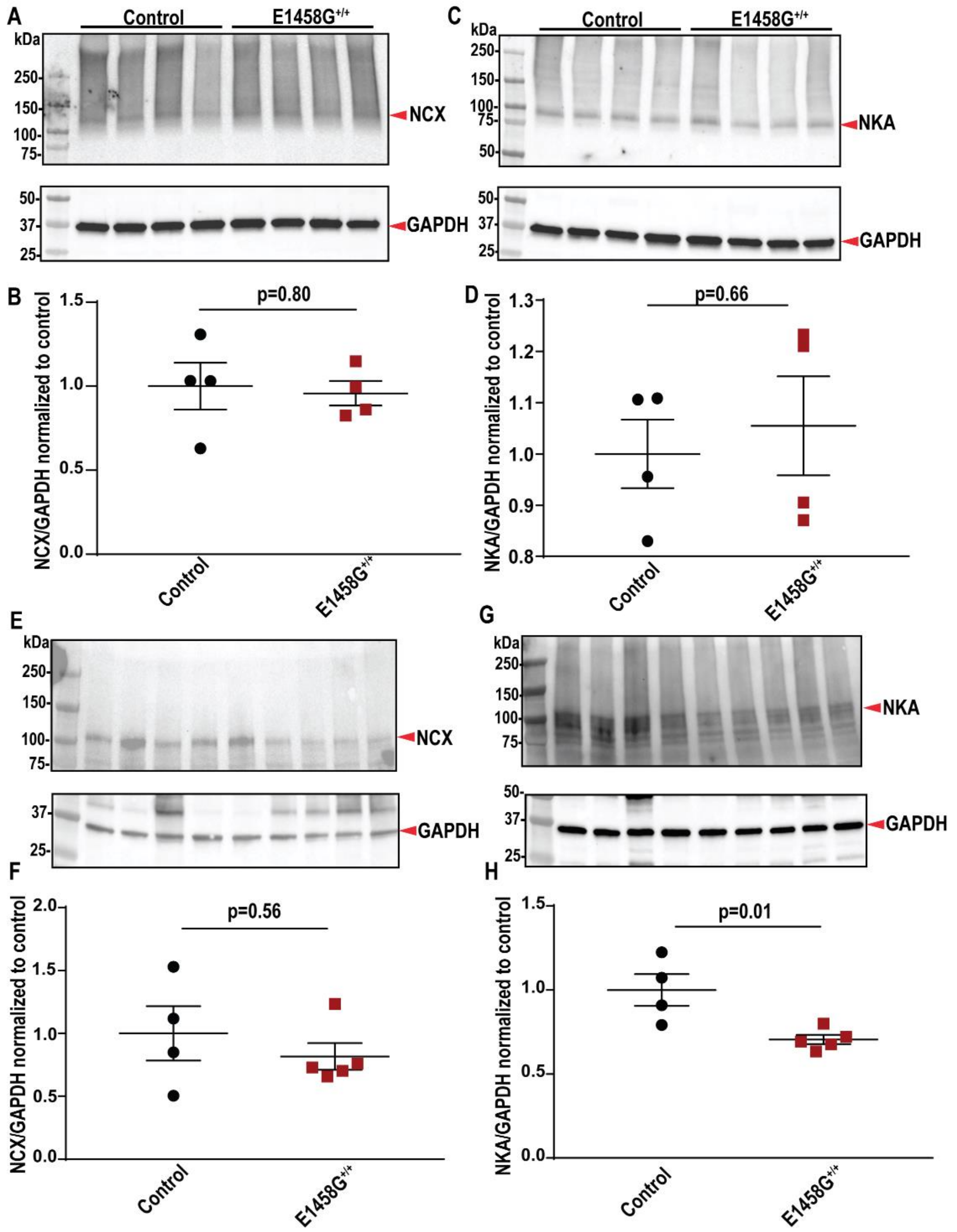

Suppl. Figure 6. Expression of ankyrin-B and ankyrin membrane partners in the AnkBp.E1458G$^{+/+}$ heart lysates in young mice at baseline (A-D) and 4 weeks post TAC (E-H). A-B. Immunoblotting and quantitative analysis of Na^+/Ca^{2+} exchanger (NCX) normalized to GAPDH. **C-D.** Immunoblotting and quantitative analysis of Na^+, K^+-ATPase (NKA) normalized to GAPDH. Data passed Shapiro-Wilk normality tests and unpaired t-test were performed (B-D). **E-F.** Immunoblotting and quantitative analysis of Na^+/Ca^{2+} exchanger (NCX) normalized to GAPDH (data did not pass Shapiro-Wilk normality test and Mann-Whitney test was performed). **G-H.** Immunoblotting and quantitative analysis of Na^+, K^+-ATPase (NKA) normalized to GAPDH (data passed Shapiro-Wilk normality test and unpaired t-test was performed). Con and AnkBp.E1458G$^{+/+}$, N=4.

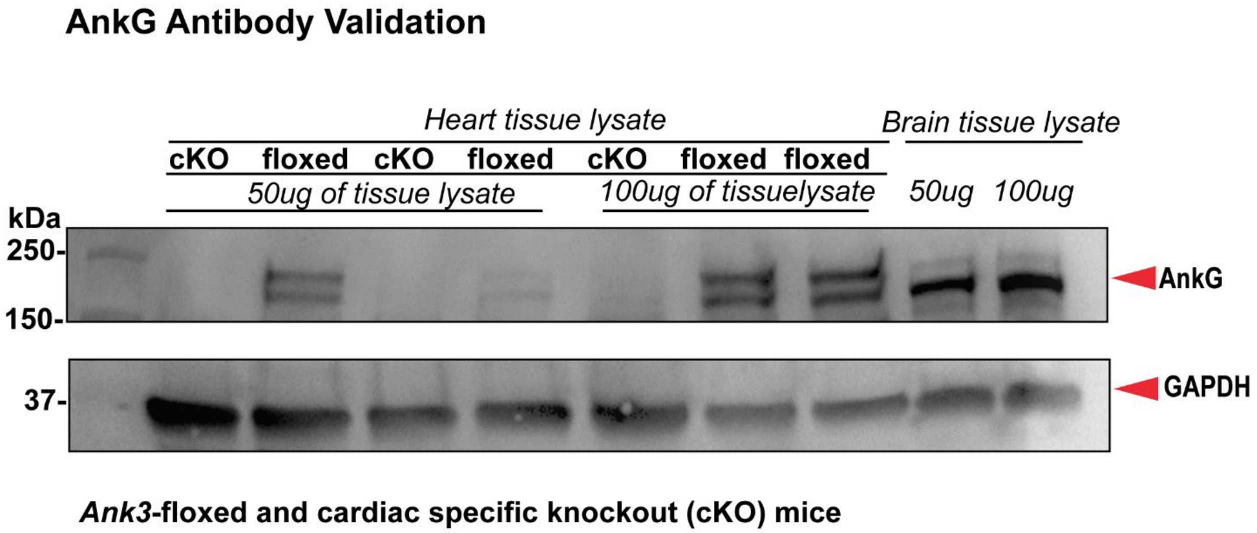

Suppl. Figure 7. Validation of AnkG antibody using floxed and *Ank3*-cardiac specific Knockout (KO).

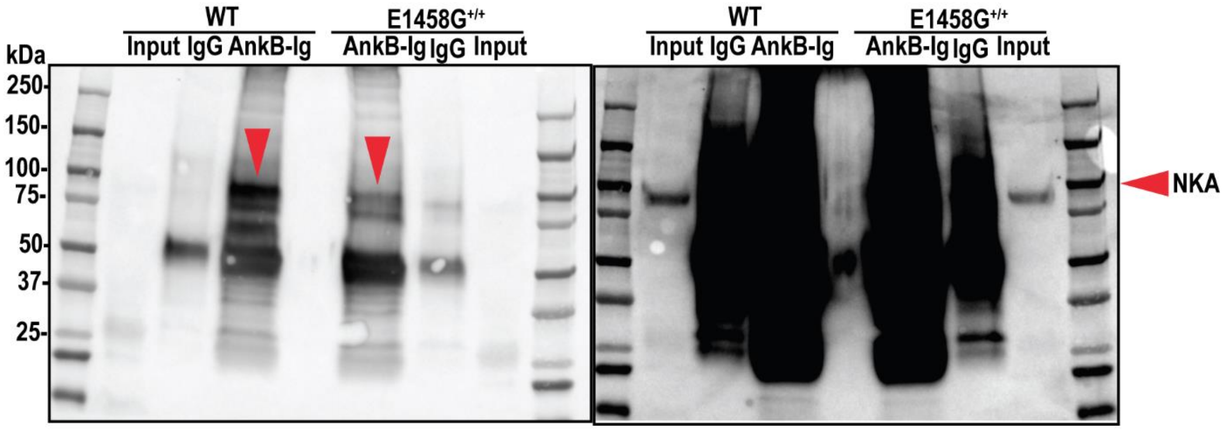

Suppl. Figure 8. Immunoprecipitation experiments illustrating the interaction between AnkB-Ig or IgG control and NKA using control and AnkBp.E1458G$^{+/+}$ lysates. Data are representative of experiments repeated three times.

Suppl. Table 1. Summary of echocardiographic parameters in control and the AnkBp.E1458G$^{+/+}$ mice at ~ six months of age.

Mouse	Ejection Fraction (%)	Fractional Shortening (%)	LVIDs (cm)	LVIDd (cm)	LVPWs (cm)	LVPWd (cm)	IVSs (cm)	IVSd (cm)
E1458G$^{+/+}$1	51.355	22.155	0.29	0.37	0.075	0.055	0.055	0.05
E1458G$^{+/+}$2	51.675	22.355	0.3	0.385	0.05	0.05	0.075	0.06
E1458G$^{+/+}$3	43.64	18.15	0.345	0.42	0.045	0.035	0.055	0.035
E1458G$^{+/+}$4	65.985	31.25	0.265	0.385	0.065	0.045	0.065	0.045
E1458G$^{+/+}$5	48.97	20.87	0.31	0.39	0.06	0.035	0.055	0.035
E1458G$^{+/+}$6	52.19	22.585	0.27	0.35	0.05	0.045	0.055	0.04
E1458G$^{+/+}$7	56.455	25.115	0.295	0.39	0.055	0.035	0.06	0.045
E1458G$^{+/+}$8	64.195	29.975	0.26	0.37	0.07	0.045	0.06	0.045
E1458G$^{+/+}$9	53.12	23.285	0.335	0.44	0.06	0.045	0.055	0.035
E1458G$^{+/+}$10	50.79	21.9	0.32	0.41	0.055	0.04	0.05	0.045
E1458G$^{+/+}$11	50.92	21.88	0.265	0.34	0.05	0.04	0.04	0.04
Control 1	66.715	31.73	0.245	0.36	0.09	0.06	0.05	0.05
Control 2	59.595	27.005	0.27	0.375	0.05	0.03	0.06	0.04
Control 3	63.62	29.705	0.295	0.425	0.06	0.04	0.07	0.055
Control 4	65.12	30.725	0.285	0.415	0.08	0.045	0.08	0.05
Control 5	56.205	24.86	0.26	0.345	0.05	0.045	0.055	0.045
Control 6	55.73	24.665	0.29	0.385	0.07	0.05	0.065	0.035
Control 7	65.83	31.09	0.255	0.375	0.075	0.05	0.06	0.04

Suppl. Table 2. Summary of electrocardiographic parameters using implanted radio-telemeters in control and the AnkBp.E1458G$^{+/+}$ mice around six months of age.

Mouse	Min HR (BPM)	Max HR (BPM)	RR Interval (S)	HR (BPM)	PR Interval (S)	P Duration (S)	QRS Interval (S)	QT Interval (S)	QTc (S)
E1458G$^{+/+}$1	596.1	678.6	0.09562	627.9	0.03699	0.008297	0.00962	0.02357	0.02412
E1458G$^{+/+}$2	503.1	663.9	0.1062	568	0.03635	0.009818	0.009136	0.02326	0.02262
E1458G$^{+/+}$3	463.9	577.9	0.1191	505.6	0.03878	0.01005	0.009507	0.02065	0.01897
E1458G$^{+/+}$4	467.2	631.5	0.1105	545	0.03449	0.008551	0.01122	0.02524	0.02391
E1458G$^{+/+}$5	420.7	623.4	0.1275	473.4	0.03608	0.009651	0.01206	0.02509	0.02224
E1458G$^{+/+}$6	527	716.7	0.09559	630	0.03547	0.009153	0.008932	0.02064	0.02116
E1458G$^{+/+}$7	498.4	700.7	0.1014	596.9	0.032	0.01118	0.009545	0.01978	0.01975
E1458G$^{+/+}$8	527.1	759.8	0.0862	700.3	0.0327	0.008767	0.009745	0.02432	0.02635
E1458G$^{+/+}$9	361.1	736.8	0.09929	612.9	0.02713	0.00507	0.01076	0.02462	0.02478
E1458G$^{+/+}$10	577.6	706.5	0.09337	644.3	0.03233	0.008422	0.009906	0.02347	0.02433
Control 1	599.2	724.3	0.08939	672	0.03034	0.009515	0.009764	0.0212	0.02243
Control 2	420.6	669.2	0.1032	590.1	0.03342	0.00959	0.009573	0.02237	0.02221
Control 3	530.8	775.1	0.08389	717.9	0.03063	0.007569	0.008603	0.02102	0.023
Control 4	573.7	722.7	0.08965	670.6	0.03191	0.008708	0.009684	0.01716	0.01818
Control 5	602.6	743.6	0.09042	665.2	0.03293	0.008028	0.009291	0.02106	0.02218
Control 6	479.1	712.5	0.09673	626.6	0.03455	0.007702	0.007723	0.02193	0.02241
Control 7	551.6	757.7	0.09011	668.8	0.03038	0.0085	0.01287	0.01948	0.02065
